# Multipurpose Iron-Chelating Ligands Inspired by Bioavailable Molecules

**DOI:** 10.3390/biom14010092

**Published:** 2024-01-11

**Authors:** Elena Cini, Guido Crisponi, Alessandra Fantasia, Rosita Cappai, Sofia Siciliano, Giuseppe Di Florio, Valeria M. Nurchi, Maddalena Corsini

**Affiliations:** 1 Department of Biotechnology, Chemistry and Pharmacy, University of Siena, Via Aldo Moro 2, 53100 Siena, Italy; elena.cini@unisi.it (E.C.); sofia.siciliano@student.unisi.it (S.S.); giuseppe.diflorio@unisi.it (G.D.F.); 2 Dipartimento di Scienze della Vita e dell’Ambiente, Cittadella Universitaria, 09042 Monserrato-Cagliari, Italy; guidocrisponi@gmail.com (G.C.); alessandra.fantasia@unica.it (A.F.); 3 Dipartimento di Scienze Chimiche, Fisiche, Matematiche e Naturali, University of Sassari, Via Vienna 2, 07100 Sassari, Italy; rcappai1@uniss.it

**Keywords:** iron, maltol, iron chelator, polyphenols, hydroxypyrones, pyrogallol

## Abstract

Because of their capacity to bind metals, metal chelators are primarily employed for therapeutic purposes, but they can also find applications as colorimetric reagents and cleaning solutions as well as in soil remediation, electroplating, waste treatment, and so on. For instance, iron-chelation therapy, which is used to treat iron-overload disorders, involves removing excess iron from the blood through the use of particular molecules, like deferoxamine, that have the ability to chelate the metal. The creation of bioinspired and biodegradable chelating agents is a crucial objective that draws inspiration from natural products. In this context, starting from bioavailable molecules such as maltol and pyrogallol, new molecules have been synthetized and characterized by potentiometry, infrared spectroscopy and cyclic voltammetry. Finally, the ability of these to bind iron has been investigated, and the stability constants of ferric complexes are measured using spectrophotometry. These compounds offer intriguing scaffolds for an innovative class of versatile, multipurpose chelating agents.

## 1. Introduction

It is well known that iron is crucial for normal cell and organismal function, although its reactions with active oxygen species are extremely dangerous [1]. For this reason, iron is tightly controlled through different mechanisms, pathways and proteins, which are involved in its uptake, distribution, utilization, recycling and excretion [2,3]. The accumulation of iron is linked with acute and severe clinical conditions, for example iron deficiency anemia, hemoglobinopathies and idiopathic hemochromatosis. Therefore, it is of great interest to understand how iron can be manipulated to achieve therapeutic effects [4].

In the frame of our research on iron-chelating agents, we mainly considered Fe^3+^ decorporation from organisms in iron overload situations [5,6,7]. Different applications of iron chelators are nevertheless of great importance in soil remediation, [8] colorimetric reagents [9] and the treatment of iron deficiency [10]. The requirements of the iron chelators, widely illustrated for the chelating agents to be used in iron overload treatments [5], are necessarily different according to the aims of their use.

There are numerous natural methods for the removal of iron. As example, the microorganism uses a well-defined iron acquisition strategy, which involves the synthesis of siderophores (from the Greek: “iron carriers”) which are low molecular weight chelating molecules that solubilize and transport ferric ions in aqueous media. Enterobactin is an example of a catechol-based, macrocyclic siderophore which can be isolated from several enteric bacteria [11]. Through its three catecholate moieties, enterobactin binds ferric ions forming thermodynamically stable and kinetically labile chelates [12]. This cation binding ability has a marked pH sensitivity because of the high charge density associated with high affinity for protons (pKa values, 12.1 and 8.4) [13].

Also, hydroxypyrones are frequently present in natural products, and they represent a versatile scaffold that is structurally modifiable to obtain derivatives with good biocompatibility and low toxicity profiles. Maltol (3-hydroxy-2-methyl-4-pyrone) is one of the best studied compounds of this class. It has the odor of caramel and is used as a flavor enhancer in the food, cosmetics, and pharmaceutical industries. Maltol is known as an iron-chelating agent (its corresponding complex is an approved drug in the treatment of iron deficiency anemia [14]), and also its analogues are used in the wider scenario of metal chelators [15,16]. For instance, the 3-methoxy-2-methyl-4-pyrone has been discovered as a metabolite from *Penicillium citrinum* [17] in addition to being employed as a chemical modification to maltol to inhibit metal coordination [18].

Due to all these factors, bioavailable molecules such as pyrogallol, maltol and its derivative 3-methoxy-2-methyl-4-pyrone are chosen as building blocks in the attempt to synthetize new ligands, which require both a high Fe^3+^ affinity and low toxicity profile. The synthesis of new compounds containing the hydroxypyrones moiety and a potentiometric and spectrophotometric study of Fe^3+^ complex formation are presented. The cyclic voltammetric characterization of the ligands and the corresponding ferric complexes is also discussed.

## 2. Materials and Methods

### 2.1. Reagents

For the synthesis, all reagents were purchased from commercial suppliers Sigma Aldrich, Merck, Darmstadt, Germany, VWR and used without further purification. Solvents were dried and purified by conventional methods [18] prior to use or, if available, purchased in anhydrous form.

For solution studies, NaOH, NaCl, HCl, and FeCl_3_·6H_2_O were purchased from Sigma Aldrich and used without any further purification. Fe^3+^ standard solution was prepared by dissolving the required amount of chloride salt in pure double-distilled water, acidified with a stoichiometric amount of HCl to prevent hydrolysis and standardized by the spectrophotometric method [9]. Sodium hydroxide solution was standardized by potassium hydrogen phthalate, and soda lime traps were used to prevent the carbonation process.

### 2.2. Synthesis

The reactions were carried out in oven-dried vessels and monitored by thin layer chromatography (TLC) using Merck aluminum-backed plates pre-coated with silica gel 60 (UV254). Flash column chromatography purifications were performed with Merck silica gel 60, 0.040–0.063 mm (230–400 mesh). NMR spectra were recorded at 25 °C with Brucker Advance NMR spectrometers, 400 MHz and 600 MHz for ^1^H and 101 MHz for ^13^C, using deuterated chloroform and methanol as solvents. The used solvent is specified for each spectrum presented in the supplementary information. Chemical shift values (δ) are given in parts per million (ppm) from internal reference tetramethyl–silane (TMS) and relative to the resonance of their respective residual solvent peaks (MeOD δ 3.31 for ^1^H and δ 49.00 for ^13^C). Coupling constants (J) are given in Hertz, and splitting patterns are designated as s, singlet; d, doublet; t, triplet; q, quartet; m, multiplet; bs, broad singlet. ES-MS analysis was performed with the chromatographic LC/MSD system Agilent 1100 series connected with a UV detector (254 nm) with a flow of 0.4 mL/min, 95% MeOH, 5% H_2_O, direct injection, ESI ionization, flow of the drying gas (N_2_) 9 mL/min, temperature 350 °C, atomizing pressure 40 PSI, and fragmentation 0.

Attenuated Total Reflection Fourier Transform Infrared (ATR-FTIR) spectra were collected in the 650–4000 cm^−1^ range with an Agilent Cary 630 FTIR Spectrometer equipped with a type IIa synthetic diamond crystal, single-reflection ATR accessory. All sample and background spectra were recorded with 128 scans at 4 cm^−1^ resolution.

The structures and the symbols of all compounds used in the synthesis reported below are shown in Scheme 1; the ^1^H and ^13^C NMR spectra of compounds **3**, **4**, **5**, **6** and **7** are reported in Figures S1–S10.

**2-((3-hydroxy-4-oxo-4H-pyran-2-yl)methyl)malonic acid** (**3**). Diethyl malonate **2** (150 mg, 0.30 mmol) was solubilized in a mixture of H_2_O/THF/EtOH (1:1:1) (6 mL) and LiOH. H_2_O (63 mg, 1.50 mmol) was added. The corresponding solution was stirred at room temperature for 16 h. The solvent was removed under vacuum and the reaction mixture was passed through DOWEX 50WX2 ion-exchange acidic resin (3 g). The lithium salt of product was eluted from resin with H_2_O. The product was obtained by flash chromatographic purification on silica gel eluting with 5% of MeOH in CH_2_Cl_2_ as a white solid (55 mg, 0.24 mmol, 80% yield).

ESI-MS (*m*/*z*): 229 [M + H]^+^; 251 [M + Na]^+^; 267 [M + K]^+^.

^1^H NMR (600 MHz, MeOD) δ (ppm): 7.85 (d, J = 5.4 Hz, 1H), 6.27 (d, J = 5.4 Hz, 1H), 3.12–3.06 (m, 3H).

^13^C NMR (151 MHz, MeOD) δ (ppm): 174.23, 171.99, 155.67, 151.16, 143.42, 113.34, 48.61, 27.66.

**2-((2,6-dihydroxyphenoxy)methyl)-3-methoxy-4H-pyran-4-one** (**4**). Under N_2_ atmosphere, pyrogallol (150 mg, 1.19 mmol), K_2_CO_3_ (493 mg, 3.57 mmol) and KI (198 mg, 1.19 mmol) in dry acetone (10 mL) were mixed for 20 min. Then, 2-(bromomethyl)-3-methoxy-4H-pyran-4-one (**1**, 778 mg, 3.57 mmol) [19] was added, and the reaction was carried out to reflux for 16 h. Acetone was evaporated, and the crude was solubilized in EtOAc (5 mL), washed with H_2_O (2 × 10 mL) and brine (10 mL) and the organic phases were dried over anhydrous Na_2_SO_4_, filtered, and evaporated under reduced pressure. The reaction mixture was purified by flash chromatography on silica gel eluting 0–5% gradient of MeOH in CH_2_Cl_2_. The compound was recovered as clear oil (204 mg, 0.77 mmol, 65% yield).

ESI-MS (*m*/*z*): 265 [M + H]^+^; 287 [M + Na]^+^.

^1^H NMR (400 MHz, MeOD) δ (ppm): 8.00 (d, J = 5.6 Hz, 1H), 6.70 (t, J = 8.2 Hz, 1H), 6.39 (d, J = 5.6 Hz, 1H), 6.31 (d, J = 8.2 Hz, 2H), 5.03 (s, 2H), 3.52 (s, 3H).

^13^C NMR (101 MHz, MeOD) δ (ppm): 176.54, 157.72, 155.82, 150.91, 146.77, 133.65, 124.28, 116.35, 107.28, 65.16, 59.59.

IR: 3510; 1638; 1331; 1263; 1181; 1026; 996 cm^−1^.

**2-ethoxybenzo[d][1,3]dioxol-4-ol** (**5**). Under N_2_ atmosphere, Amberlyst^®^15 (30 mg) was suspended in dry toluene (35 mL). After 30 min, pyrogallol (300 mg, 2.38 mmol) and triethyl orthoformate (1.19 mL, 7.14 mmol) were added at room temperature, and then the mixture was heated to reflux for 16 h. The reaction mixture was cooled to room temperature, filtered on a Celite pad and toluene was evaporated under reduced pressure. The compound was purified by flash chromatography on silica gel eluting with gradient 0–50% of EtOAc in petroleum ether; it was obtained as a clear oil (340 mg, 1.89 mmol, 80% of yield).

ESI (*m*/*z*): 183 [M + H]^+^; 205 [M + Na]^+^.

^1^H NMR (400 MHz, MeOD) δ (ppm): 6.78 (s, 1H), 6.63 (t, J = 8.1 Hz, 1H), 6.39 (ddd, J = 23.2, 8.1, 0.8 Hz, 2H), 3.63 (q, J = 7.1 Hz, 2H), 1.13 (t, J = 7.1 Hz, 3H).

^13^C NMR (101 MHz, MeOD) δ (ppm): 147.16, 140.24, 132.85, 121.62, 118.75, 110.61, 100.02, 59.10, 14.00.

**2-(((2-ethoxybenzo[d][1,3]dioxol-4-yl)oxy)methyl)-3-methoxy-4H-pyran-4-one** (**6**). Under N_2_ atmosphere, pyrogallol orthoformate **5** (100 mg, 0.55 mmol), K_2_CO_3_ (227 mg, 1.65 mmol) and KI (91 mg, 0.55 mmol) in dry acetone (10 mL) were mixed for 20 min. Then, compound 1 (361 mg, 1.65 mmol) was added, and the reaction was carried out to reflux for 16 h. Acetone was evaporated, and the crude was solubilized in EtOAc (5 mL), washed with H_2_O (2 × 10 mL) and brine (10 mL), and the organic phases were dried over anhydrous Na_2_SO_4_, filtered, and evaporated under reduced pressure. The alkylated product was purified by means of chromatography on silica gel with ISOLERA Biotage^®^ eluting 0–60% gradient of EtOAc in petroleum ether. Compound 3 was recovered as a clear oil (90 mg, 0.34 mmol, 65% yield).

ESI-MS (*m*/*z*): 321 [M + H]^+^; 343 [M + Na]^+^.

^1^H NMR (400 MHz, MeOD) δ (ppm): 8.05 (d, J = 5.6 Hz, 1H), 6.92 (s, 1H), 6.83 (dd, J = 8.5, 7.9 Hz, 1H), 6.65 (ddd, J = 24.7, 8.2, 1.0 Hz, 2H), 6.46 (d, J = 5.6 Hz, 1H), 5.18 (s, 2H), 3.81 (s, 3H), 3.72 (q, J = 7.1 Hz, 2H), 1.23 (t, J = 7.1 Hz, 3H).

^13^C NMR (101 MHz, MeOD) δ 176.15, 156.37, 155.77, 147.52, 146.70, 141.40, 134.76, 121.78, 119.23, 116.65, 110.42, 102.78, 63.72, 60.01, 59.30, 13.61.

**2-((2,3-dihydroxyphenoxy)methyl)-3-methoxy-4H-pyran-4-one** (**7**). Compound **6** (50 mg, 0.16 mmol) was solubilized in MeOH (1 mL), and HCl 3 M in MeOH (50 µL, 0.16 mmol) drop by drop at 0 °C was added. Then, the solution was stirred at room temperature for 2 h. The solvent was removed under vacuum, and the reaction mixture was purified by flash chromatography on silica gel eluting 0–5% gradient of MeOH in CH_2_Cl_2_. Compound **7** was recovered as a white solid (40 mg, 0.15 mmol, 97% yield).

ESI-MS (*m*/*z*): 265 [M + H]^+^; 287 [M + Na]^+^.

^1^H NMR (400 MHz, MeOD) δ (ppm): 8.02 (d, J = 5.6 Hz, 1H), 6.63–6.55 (m, 1H), 6.49 (td, J = 7.8, 1.5 Hz, 2H), 6.42 (d, J = 5.6 Hz, 1H), 5.07 (s, 2H), 3.76 (s, 3H).

^13^C NMR (151 MHz, MeOD) δ 176.36, 157.10, 155.85, 146.66, 146.10, 135.38, 118.54, 116.53, 109.94, 106.76, 63.34, 59.92.

IR: 3452; 1645; 1458; 1308; 1080 cm^−1^.

### 2.3. Solution Equilibrium Studies

Protonation and complex formation equilibria were studied at 25 °C and 0.1 M NaCl ionic strength by combined potentiometric–spectrophotometric titrations at ligand concentrations ranging from 5 × 10^−4^ M to 1 × 10^−3^ M and different metal:ligand molar ratios. Potentiometric measurements, managed by Metrohm TiAMO 1.2 software, were performed with a dEcotrode plus Metrohm combined glass electrode connected to 888 Titrando (Metrohm AG, Herisau, Switzerland). The electrode was daily calibrated for hydrogen ion concentration by HCl standard titration with NaOH in the used experimental conditions, and data were analyzed by Gran’s method [20]. Spectrophotometric measurements were performed in the 200–360 nm and 360–800 nm wavelength range for protonation and complex formation equilibria, respectively, using a 0.2 cm or 1.0 cm fiber optic dip probe connected to an Agilent Cary 60 UV-Vis spectrophotometer operated by Varian Cary WinUV software. Potentiometric and spectrophotometric data were processed by HyperQuad [21] and HypSpec [22] programs, respectively. Log β_pqr_ values are related to the overall equilibria pM + qH + rL ⇆ M_p_H_q_L_r_ where electrical charges are omitted. L refers to the completely deprotonated form of the ligands (L^3−^ for **3** ligand and L^2−^ for **4** and **7** ligands). The hydrolysis constants of Fe^3+^ at 25 °C and 0.1 M ionic strength logβ_1–1_ = −2.563, logβ_1–2_ = −6.205, logβ_1–3_ = −12.497, logβ_1–4_ = −21.883, logβ_2–2_ = −2.843 and logβ_3–4_ = −6.054 [23] were taken into account for the calculations of complex formation constants.

One the most useful parameters for the evaluation of the binding ability of a ligand toward a given metal ion is pM, which is defined as −log [M^n+^] free at [ligand] = 10 μM and [M^n+^] = 1 μM at pH = 7.4 [24].

### 2.4. Cyclic Voltammetry Studies

Cyclic voltammetry studies were carried out in buffer phosphate 0.1 M (pH 7.4), using a three-electrode configuration (glassy carbon working electrode, Pt counter electrode, Ag/AgCl (KCl, 3M) reference electrode) at a BAS100W potentiostat. The working electrode was polished mechanically for 2 min using 0.05 μm Alumina polishes followed by flushing with deionized water for a few minutes. Then, it was sonicated for 10 min. The polished electrode was then electrochemically tested using a solution of 2 mM potassium hexacyanoferrate solution containing KCl 1 M between −0.2 and 0.6 V vs. Ag/AgCl (+0.46 V). All the potential values reported are referred to CV at 200 mV/s.

## 3. Results

### 3.1. Synthesis and ATR-Characterization

**Scheme 1:**
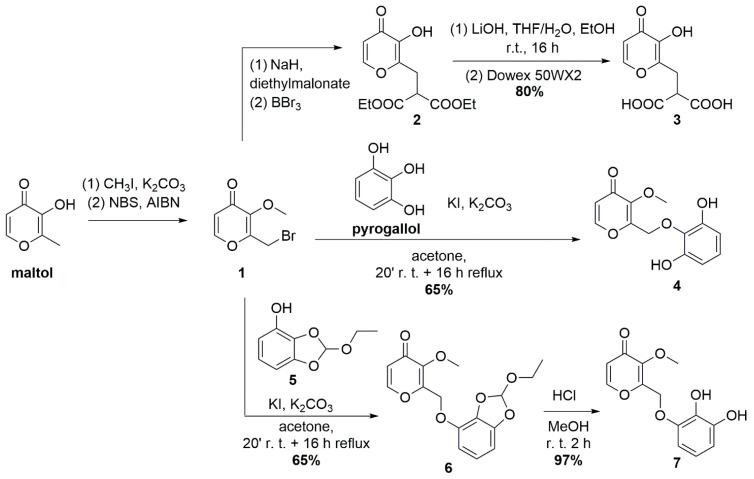
Synthesis of compounds **3**, **4** and **7**. Compounds **1** and **2** were prepared as described in the literature [19,26].

In Scheme 1, we report the synthetic pathway for derivatives **3**, **4** and **7**. Compound **3** was obtained by basic hydrolysis of compound **2** and subsequent treatment with acidic Dowex resin. Catechol **4** was synthesized by the direct alkylation of resorcinol with bromide **1**, which was prepared as described in the literature [19]. To obtain the regioisomer **7**, the protection of vicinal -OH groups of pyrogallol was required. Orthoester **5** was prepared from pyrogallol in the presence of triethyl orthoformate under the conditions applied to ethyl gallate protection [25] and then alkylated at the free hydroxy group with K_2_CO_3_ as base; final deprotection by acid treatment gave compound **7**.

**Figure 1:**
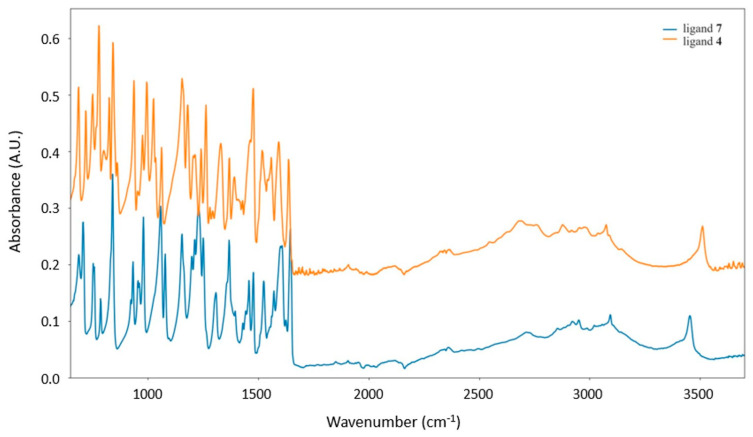
ATR-IR spectra of isomers **4** and **7**. The baseline of ligand **4** is uplifted for clarity.

Infrared-ATR spectra were recorded to distinguish between the regioisomers **4** and **7**. The collected spectra are presented in Figure 1. In the region of OH-stretching, the ligands **4** and **7** show peaks at 3510 and 3452 cm^−1^, respectively. The shift of this vibration depends on the different respective positions in the ring of the two OH groups.

The methoxy-maltol subunit shows a doublet at 1618 and 1640 cm^−1^ that can be attributed to carbonyl stretch (ν_C=O_) and carbon double bonds of the maltol ring (ν_C=C_) [18,27]. Moreover, pyrogallol shows ring carbon–carbon double bonds at 1618, 1517 and 1482 cm^−1^ [28]. Carbon double bonds of the maltol ring are present in both **4** and **7** with a small blue shift (1645 cm^−1^) for **7**. A band at around 1600 cm^−1^ is observed in both **4** and **7**, which is probably the result of the overlap of the maltol carbonyl stretch and pyrogallol ring vibration [27,28]. A band of pyrogallol at 1482 cm^−1^ is present in **7** and **4**; however, a band at 1458 cm^−1^ is evident in the spectrum of **7** and it is only a shoulder in the spectrum of **4**. Similarly, around 1360 cm^−1^, one band is present for the **4** spectrum, which becomes a doublet for **7**. The spectrum of methoxy-maltol shows peaks at 1360 and 1448 cm^−1^, belonging to the symmetric and asymmetric bending of methyl (δ_CH3_) [18]. Thus, differences observed at 1360 and 1458 cm^−1^ between **7** and **4** may depend on different molecular structures. The different molecular arrangements in the two compounds might interfere with the bending of CH_3_ and, in case of **4**, reduce its intensity.

Both compounds show two peaks at 1370 and 1331 cm^−1^ (lowered to 1308 cm^−1^ for **7**) of uncertain attribution. Tentatively, here, pyrogallol has a band (1364 cm^−1^) involving in-plane OH bending and other vibrations and a band (1323 cm^−1^) involving ν_CO_ [28]. Of course, the peak position (1308 cm^−1^ for **7** and 1331 cm^−1^ for **4**) may be influenced by the COC reaction site.

In the region 1150–1300 cm^−1^, methoxy-maltol shows strong peaks at 1163, 1208, 1246 cm^−1^ [18] mainly attributed to CH bending (δ_CH_), and pyrogallol has an intense vibration at 1185 cm^−1^ due to in-plane OH bending and CC stretching [28]. The two studied compounds show an intricate spectrum in this region with several modifications. Nevertheless, it is possible to mark the difference in a peak at 1181 cm^−1^ present only in the **4** spectrum and a shift to 1263 cm^−1^ of a supposedly CH bending peak for **4**. Alternatively, one of these bands could be related to the C-O-C vibration between pyrogallol and methoxy-maltol [29].

In the low wavenumber region falls the wagging (γ_CH_ 824 cm^−1^) vibration of methoxy-maltol [30], which is probably shifted around 840 cm^−1^ for both the **7** and **4** compounds. Alternatively, the strong peak at 840 cm^−1^ might be assigned to the C-O-C linkage between subunits. Furthermore, methoxy-maltol presents at 916 and 986 cm^−1^ the symmetric (ν_s_) and antisymmetric stretch (ν_as_) of C-O-C in the ring [18,30]. These bands may probably be found in **7** and **4** around the 930 and 980 cm^−1^ range; however, **4** shows two more peaks at 996 and 1026 cm^−1^. At 995 cm^−1^, pyrogallol has a vibration that involves the stretching of CC, CO and bending of CO [28]. Interestingly, **7** does not show a similar peak, or it is heavily shifted. In this region, however, **4** and **7** show several infrared features of difficult assignment and, most probably, also bands related to the C-O-C bond, linking the two subunits, fall in this part of the spectrum.

### 3.2. Protonation Equilibria

The protonation equilibria of the three ligands **3**. **4** and **7** presented in Scheme 1 were studied by joined potentiometric–spectrophotometric titrations (Figures S11–S21) thanks to the spectral UV variations observed during the protonation of the 3-hydroxy group in **3** and of the aromatic OH groups in **4** and **7**. The protonation of the carboxylic groups in **3** is instead spectral silent, so their protonation constants were determined from the potentiometric data. The protonation constants of the three ligands are reported in Table 1.

**Figure 2:**
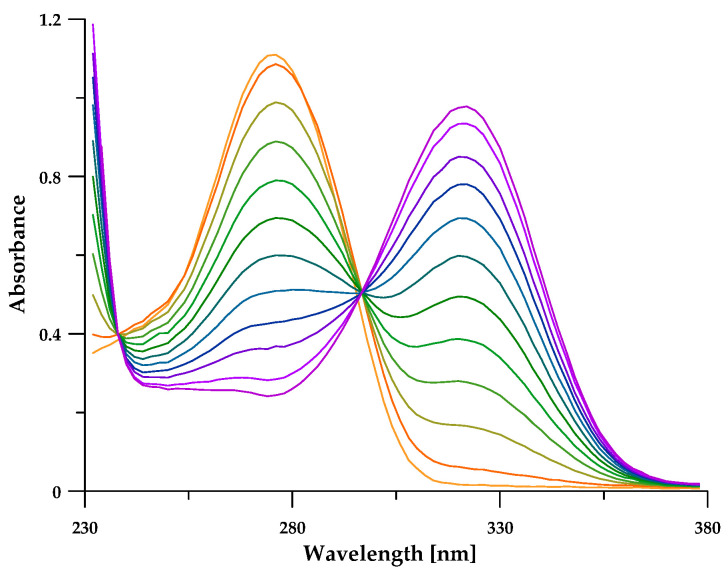
Representative spectra of ligand **3** collected during UV titration (l = 0.2 cm) at 25 °C, 0.1 NaCl ionic strength, 6.3 × 10^−4^ M ligand concentration, 234–274 wavelength range and 5.45–10.71 pH range.

Ligand **3** is characterized by three acidic groups: the hydroxy group of the maltol unit and the two carboxylic groups of the malonic acid unit. The UV spectra collected in Figure 2, processed by the HypSpec program [22], indicate that the first protonation takes place with a log *K* of about 9 with log β = 8.999 (1). The trend of absorbance vs. pH on the maxima (Figure S13) illustrates the goodness of the fit between the experimental points (dots) and the calculated trend with the optimized protonation constant. The remaining two protonation constants related to the carboxylic groups were evaluated, using the Hyperquad program [21], from the potentiometric titration (Figure S11) obtaining the log *K* values 4.87 and 2.83.

The protonation constants of **3** can be compared with that of maltol, as far as the hydroxypyrone unit is involved, and with those of malonic acid and methylmalonic acids when the carboxylic groups are concerned (Table S1).

In terms of deprotonation, log *K* 2.83 (Table 1) calculated for the first deprotonation is comparable with the literature values for methyl malonic acid [31]. The second deprotonation, 4.87, is about 0.5 units lower than the values for methyl malonic acid [32] and 0.4 than those for malonic acid [33]: the formation of a hydrogen bond between the carboxylate and the OH group of maltol unit presumably favors the easier loss of the second proton. The log *K* 8.99 (calculated for the deprotonation of maltol unit), about 0.5 units higher than the literature values for maltol [34,35], supports the stabilizing role of the hydrogen bond, which moves up the loss of a carboxylic proton and delays that of the malonic moiety. The higher negative charge of **3** with respect to simple maltol should also contribute to the increased value of log *K*.

The protonation constants of ligand **4** (Table 1) were evaluated from the spectrophotometric titration in Figure S15. Figure S17 shows the fitting of absorbance vs. pH at 230 nm and 290 nm, where the spectral variations are more remarkable.

The protonation constants (log *K*) of **4** 11.55 and 8.91, compared with the values for resorcinol in the same experimental conditions reported by Herrero-Martinez et al. [36], 11.43 and 9.36, appear very similar even if a difference of 0.45 units in the second has to be remarked. As far as the protonation constants of ligand **7** are concerned, 11.30 and 8.95, these can be compared with those of Sigel et al. [37]: 12.10 and 9.31, for 1,2,3-trihydroxybenzene-1-methyl ether. In this case, a sensible difference is apparent for the first protonation constant, and it is less marked for the second. The found differences can be tentatively explained by assuming an intramolecular hydrogen bond between the hydrogen atom of OH groups and some accepting group as the carbonyl or the -O-CH_3_ group.

The protonation constants of **7** in Table 1 were evaluated from the spectrophotometric titration in Figure S19. Figure S21 presents the fitting of absorbance vs. pH at the two wavelengths 240 nm and 290 nm. Ligand **7** can be considered as a 3-substituted catechol molecule, where the non-coordinating group is the substituent. Its action is to lower the protonation constants, log *K* 14.3 and 9.17, of unsubstituted catechol to 11.3 and 8.95. In 1,2,3-trihydroxybenzene-1-methyl ether, where the substituent is a methoxy group, the first protonation constant [37] is more than 2 units lower than that of catechol. The methoxy group, according the theory of Swain and Lupton [38], exerts a high inductive effect (+0.413) compensated by a high resonance contribute (−0.501), so it can induce either the lowering or the increase in protonation constants according to its position in the ring. At any rate, in **7**, we observe a strong decrease in the first protonation constant and a modest decrease in the second one with respect to that of pure catechol.

### 3.3. Fe^3+^ Complex Formation Equilibria

**Figure 3:**
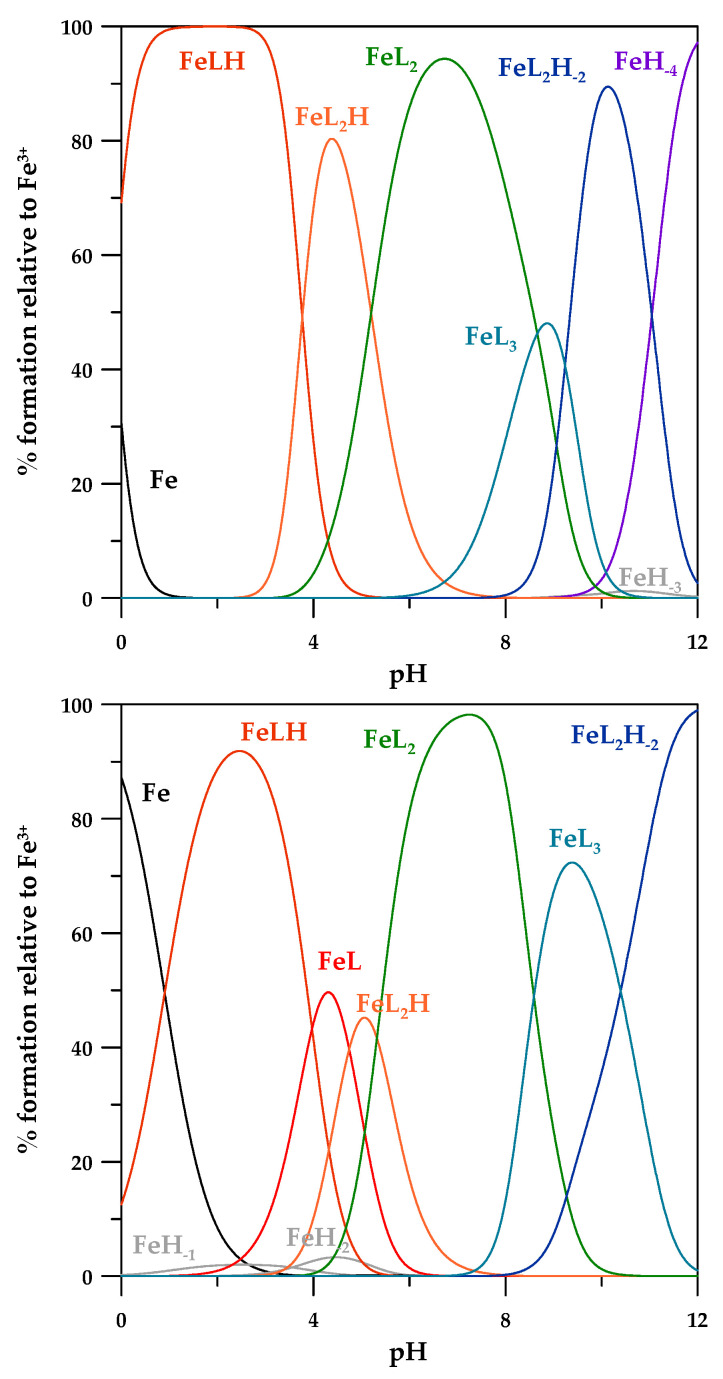
Speciation plots of Fe^3+^–ligand system (**3** up, **7** down) calculated with the HySS program [39] at 1:3 metal:ligand molar ratio and 1 mM ligand concentration.

The Fe^3+^ complex formation equilibria of **3** were studied at 1:2 and 1:3 metal:ligand molar ratios by joined potentiometric–spectrophotometric titrations. The formed complexes and the corresponding stability constants are reported in Table 2, and the related speciation plots are shown in Figure 3.

**Scheme 2:**
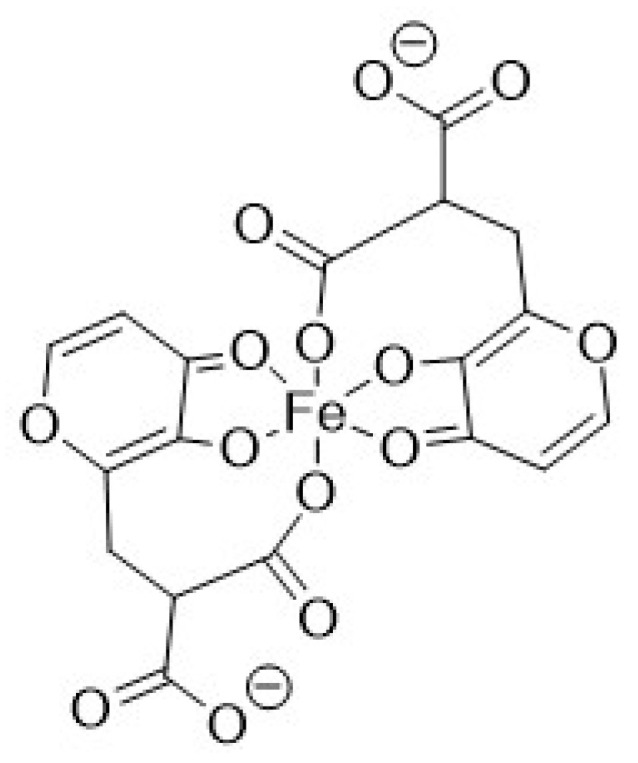
A possible structure of the [FeL_2_]^3−^ complex.

In the case of ligand **3** (Figures S22 and S23), a very strong [FeLH]^+^ complex is almost completely formed at pH 0, in which the Fe^3+^ is coordinated through the two oxygen atoms of the maltol unit. One carboxylic group has already lost its proton, two units before than in the free ligand, being presumably involved in the coordination or simply due to the high positive charge conferred by Fe^3+^ to the entire molecule. A second ligand coordinates iron at pH about 4, being only one of the four carboxylic groups until protonated. This last proton is lost with p*K* 5.20, which is slightly higher than in the free ligand (p*K* 4.87) due to the negative charge of the [FeL_2_H]^2−^ complex. In Scheme 2, a possible structure of this last complex is presented, showing the involvement of the malonic unit in iron binding.

The highly negatively charged complex, [FeL_3_]^6−^, starts to form at pH > 7, which turns into the hydroxo complex [FeL_2_H_-2_]^5−^ at pH about 9 and then decomposes in the metal hydroxide [Fe(OH)_4_]^−^ and in the free ligand L^3−^. At pH 7.4, 98% of total iron in the form of the stable [FeL_2_]^3−^ complex determines the high pFe^3+^ value 17.72. The complexation of Fe^3+^ by ligand **3** can be compared with those with the parent molecules, maltol and malonic acid, which form iron complexes with three different stoichiometries: for maltol [FeL]^2+^(log β_1_ = 11.5), [FeL_2_]^+^ (log β_2_ = 21.40) and FeL_3_ (log β_3_ = 29.7) [40], which determine a pFe^3+^ value of 16.51 being 98.7% of Fe^3+^ in the form FeL_3_; for malonic acid [FeL]^+^ (log β_1_ = 7.52), [FeL_2_]^−^ (log β_2_ = 13.29) and [FeL_3_]^3−^ (log β_3_ = 16.93) [41] that determine a pFe^3+^ value of 9.20. As stated by the authors, in these complexes, each ligand coordinates the metal ion in a bidentate way through the two deprotonated O^−^ groups. Ligand **3** is a stronger chelating agent than the parent maltol (pFe^3+^ 17.72 vs. 16.51), and above all, the pFe^3+^ value is determined by the complex [FeL_2_]^3−^. These findings lead us to hypothesize that in the complex, the malonic moieties contribute to the octahedral iron coordination.

**Figure 4:**
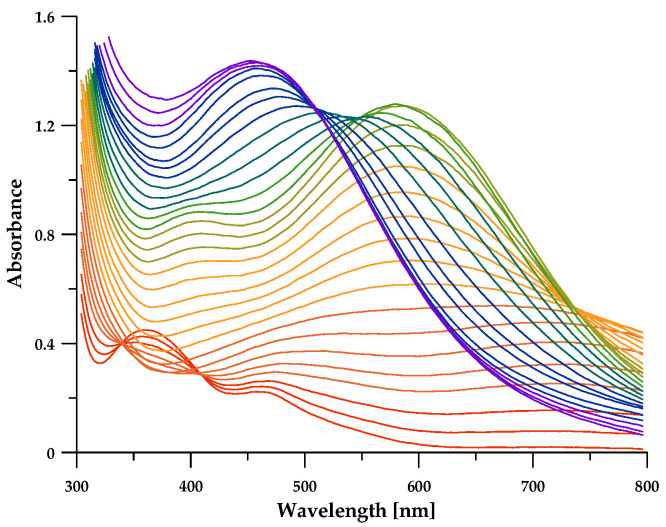
Representative spectra of Fe^3+^-**7** system at 1:3 metal:ligand molar ratio ligand collected during UV-Vis titration (l = 1 cm) at 25 °C, 0.1 NaCl ionic strength, 9.74 × 10^−4^ M ligand concentration, 300–800 wavelength range and 3.08–11.02 pH range.

In the case of **7** (Figure 4, Figures S24 and S25), despite many similarities with **3**, the formed complexes are quite different. The first complex [FeLH]^2+^ starts to form at pH 0 and reaches its maximum of formation at pH 2.2 (>90%) where Fe^3+^ is coordinated by one O^−^ group of catechol being the second until protonated. This complex transforms in the [FeL]^+^ at pH 4 where Fe^3+^ is coordinated by both oxygen atoms of the catechol group. At pH 5, the neutral complex, FeL_2_H, reaches the maximum of formation (<50%) giving the highly stable complexes, [FeL_2_]^−^ (97.8%), at pH 7.4 and losing the remaining catecholic proton. Increasing the pH, the formation of the octahedrally coordinated [FeL_3_]^3−^ is observed, which is characterized by the high negative charge of the catecholate complexes. At very basic pH, a hydroxo complex [FeL_2_H_-2_]^3−^ is formed; it has to be noted that from the spectral appearance, no disruption of the complexes takes place.

Additionally, the strong decrease in the first protonation constant and a modest decrease in the second one, with respect to that of pure catechol (discussed in Section 3.3), is accompanied by the noteworthy decrease in the iron stability constants. Consequently, as remarked in a previous work that explains the dependence of pM on the competition between proton and metal ion for the same binding sites on the ligand [42], the global effect is an increase in pFe^3+^ of more than 2 units regarding that of catechol.

### 3.4. Cyclic Voltammetry

In order to determine the redox potential of the ligands and their relative iron complexes, the electrochemical behavior of **4** and **7** compounds was investigated by cyclic voltammetry at a glassy carbon electrode in phosphate buffer (pH 7.4, 0.1 M) saturated with N_2_, and during the voltammetric measurements, a constant flow of N_2_ was kept over the solution surface in order to avoid the diffusion of atmospheric oxygen into the solution of compounds. The ligands **4** and **7** were dissolved in MeOH and then in buffer phosphate (8% *v*/*v*) to reach the concentration 3.0 × 10^−3^ M. In this experimental condition, ligand **3** undergoes an irreversible oxidation at E_pa_ = +0.87 V.

**Figure 5:**
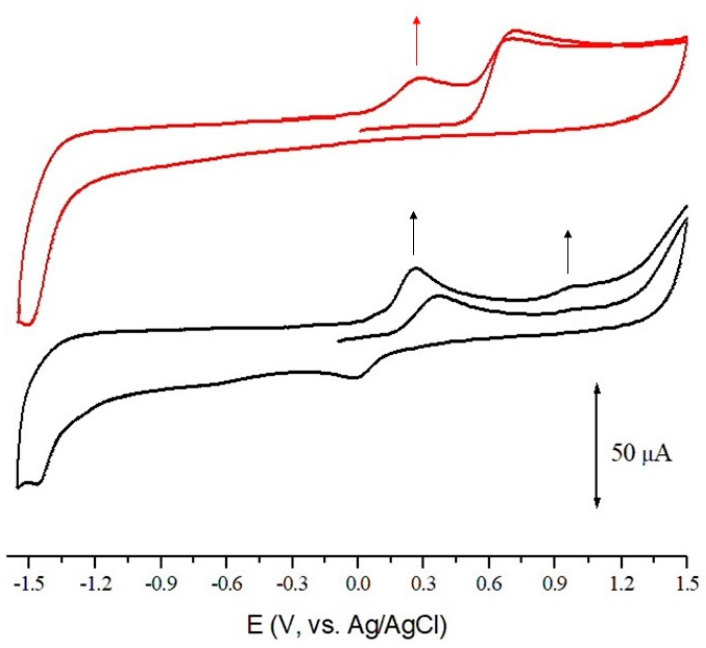
Cyclic voltammograms recorded on glassy carbon electrode on a solution 3 mM of **4** (red line) and **7** (black line) in buffer phosphate. Scan rate 200 mV/s.

The cyclic voltammograms recorded on a solution of **4** show an irreversible oxidation process (E_pa_ = +0.69 V) and, in the back positive scan, the appearance of a new irreversible oxidation peak at E_pa_ = +0.38 V (Figure 5, red line). For this last process, the calculated parameter i_pa_/√v is constant with scan rate, ruling out the possibility that it is caused by adsorption processes.

Moreover, ligand **7** undergoes a principal electrochemically quasi-reversible oxidation process (E°′ = +0.18 V; ΔE = 274 mV, i_pc_/i_pa_~1) catechol centered (Figure 5, black line). As consequence of this oxidation, the monomer **7** undergoes polymerization, and the new formed polymer oxidizes irreversibly at +0.24 V. The phenomenon of catechol electropolymerization and the resulting creation of a poly(catechol) film has been extensively researched. For additional information, consult the references [43,44,45].

The application of voltage results in the formation of a further second irreversible oxidation peak at +0.99 V, as seen by the black line in Figure 5. It is difficult to speculate on the nature of this secondary process even for the difficulty in identifying the multitude of compounds that can be formed upon the oxidation of ligand **7** or because it could be due to the oxidation of OH^−^ ions.

Cyclic voltammograms showed no new processes at any scan rate when an amount of FeCl_3_ solution (1:3 metal: ligand molar ratio) was added to the ligand **4** solution.

Otherwise, the cyclic voltammograms obtained in the Fe^3+^-**7** solution exhibit the irreversible oxidation process at +0.24 V (perhaps due to the electrodeposited polymer) as well as the monomers’ oxidation wave disappearing (Figure S26). It suggests that ligand **7** is rapidly oxidized by ferric ions to the corresponding polymer, which promptly restores the electrode surface. When this modified electrode or a polished glassy carbon electrode is used, there is no evidence of redox processes involving the expected metal complex. It is known that catechols could coordinate transition metal ions, but they also can undergo metal-catalyzed oxidation and/or crosslinking reactions. As a consequence, it is plausible that either the complex concentration is too low for cyclic voltammetry sensibility or it undergoes reduction at potentials lower than −1.5 V. Given the reported Fe^3+^-**3** reduction potential was +0.40 V [26], it is reasonable to attribute the electrode potential value shift to the different chemical moiety involved in metal coordination (maltol vs. catechol).

### 3.5. Discussion

In attempt to balance toxicity and safety, some chemical characteristics are required to iron chelators [46]. The new ligands **3**, **4** and **7** have a molecular weight less than 500 g/mol, which is the cutoff molecular weight for drugs that have to be absorbed in the human intestine [47]. Furthermore, the presence of oxygen donor atoms in the ligands suggest a specific affinity for Fe^3+^, but the catechol moiety is essential in order to guarantee the best coordination for **7** rather than for **4**. In fact, **7** possesses a higher *β*_3_ value than that of **3** for Fe^3+^, but the corresponding pFe values are lower. This difference can be due to the relatively higher affinity of catechol for protons; indeed, due to the relatively low protonation constant (pK_a_~9), at physiological pH, hydrogen ion interference is less pronounced for **3** and maltol than for that of ligands **7** and **4**.

Moreover, two possible reaction pathways can occur when ferric iron and the catechol moiety interact (both of them are pH-dependent): through redox reactions, which involve oxidizing 1,2-dihydroxybenzene to produce quinones that polymerize, or through complexation reactions, which involve the formation of a coordination bond between Fe^3+^ and deprotonated catechol. In the experimental condition of the present work (pH 7.4), peak potential values of the oxidation process (E_pa_) of the three ligands appear to reflect the tendency toward a preference for oxidation reactions over complexation reactions, in particular, coordination reactions are favored when E_pa_ increases (**7** < **4** < **3**). Given all of these aspects, ligand **3** seems to be the most promising chelating agent at physiological pH.

## 4. Conclusions

The three new ligands **3**, **4** and **7** have been synthesized and characterized for their acid–base behavior and, in particular, for their complex formation equilibria with Fe^3+^. Molecule **4** does not form complexes of noticeable stability, and the topic was not explored further. The other two ligands form Fe^3+^ complexes of strong stability. The **3** complex presents interesting features: mainly the involvement of the malonate group in coordination. The ligand **7** complexes show on their behalf the strong role of the non-binding unit as a substituent of the catechol moiety that increases the pFe^3+^ of **7** by more than 2 units with respect to that of catechol.

Therefore, further efforts will be made to analyze the demethylation of the maltol moiety.

Moreover, cyclic voltammetry measurements confirm the higher affinity of ligand **7** for Fe^3+^ to respect Fe^2+^ and suggest that this ligand could remove free iron from the Fenton reaction in physiological conditions.

To conclude, these complexes could be considered for their possible applications in particular as iron-chelating agents, as colorimetric reagents and as iron suppliers. The found pFe^3+^ values of both ligands, 17.72 and 16.86 for **3** and **7**, respectively, prevent their use as iron chelators, since they cannot extract the iron bound to transferrin, so they cannot exert a scavenging action in human organisms. Moreover, the possible application as colorimetric reagents of ligands **3** and **7** is somewhat hindered. While they satisfy the majority of requisites of a good color reagent outlined in a recent paper [9], i.e., high stability of complexes, the fast reaction of complex formation, and high values of absorptivity, they fail in the formation of a complex that has definite stoichiometry stability across a wide range of pH values. The speciation plots of both ligands (Figure 3) show the formation with pH of largely overlapping complexes of different stoichiometry, each characterized by a definite individual spectrum.

The values of stability constants are instead indicative for a possible utilization of the iron complexes of **3** and **7** as iron supplements or as iron-fortifying molecules for food and drinks [10], which requires a study of different properties such as absorption, toxicity and taste by skilled biomedical researchers.

## Figures and Tables

**Table 1:** Protonation constants of the three studied ligands evaluated from potentiometric and spectrophotometric titrations at 25 °C and 0.1 M NaCl ionic strength reported as log β and the corresponding log *K*.

	3			4		7	
species	log β	log *K*	species	log β	log *K*	log β	log *K*
[LH]^2−^	8.999 (1)	8.999	[LH]^−^	11.55 (2)	11.55	11.30 (6)	11.30
[LH_2_]^−^	13.87 (4)	4.87	LH_2_	20.46 (2)	8.91	20.25 (3)	8.95
LH_3_	16.70 (5)	2.83					

**Table 2:** Complex formation constants of Fe^3+^ with **3** and **7** ligands evaluated from combined potentiometric–UV titrations at 25 °C, 0.1 M NaCl ionic strength.

	3			7	
Species	log β	p*K*	Species	log β	p*K*
[FeLH]^+^	20.39 (1)		[FeLH]^2+^	22.44 (1)	3.97
[FeL_2_H]^2−^	30.33 (1)	5.20	[FeL]^+^	18.47 (2)	
[FeL_2_]^3−^	25.13 (1)	9.06	FeL_2_H	37.29 (1)	5.32
[FeL_2_H_-2_]^5−^	7.01 (2)		[FeL_2_]^−^	31.97 (1)	9.27
[FeL_3_]^6−^	29.60 (3)		[FeL_2_H_-2_]^3−^	13.43 (2)	
			[FeL_3_]^3−^	39.03 (1)	
pFe^3+^	17.72			16.86	

## Data Availability

The original data can be requested to authors.

## Supplementary Materials

The following supporting information can be downloaded at https://www.mdpi.com/article/10.3390/biom14010092/s1.
