# Supplementary material for: Multipurpose Iron-Chelating Ligands Inspired by Bioavailable Molecules
Source: Biomolecules. 2024 Jan 11;14(1):92. doi: 10.3390/biom14010092 (PMC10813012; doi:10.3390/biom14010092)
Supplement: Supplementary file 1 [file biomolecules-14-00092-s001.zip › biomolecules-2805445-supplementary.pdf]

Multipurpose iron-chelating ligands inspired by bioavailable molecules

Elena Cini [1], Guido Crisponi [2], Alessandra Fantasia [2], Rosita Cappai [3], Sofia Siciliano [1], Giuseppe Di Florio [1], Valeria M. Nurchi [2,*], Maddalena Corsini [1,*]

[1] Department of Biotechnology, Chemistry and Pharmacy, University of Siena, via Aldo Moro 2, 53100 Siena, Italy; elena.cini@unisi (E.C.), sofia.siciliano@student.unisi.it (S.S.); giuseppe.diflorio@unisi.it (G.D.F.)

[2] Dipartimento di Scienze della Vita e dell'Ambiente, Cittadella Universitaria, 09042, Monserrato-Cagliari, Italy; alessandra.fantasia@unica.it (A.F.); guidocrisponi@gmail.com (G.C.); nurchi@unica.it (V.M.N.)

[3] Dipartimento di Scienze Chimiche, Fisiche, Matematiche e Naturali, University of Sassari, via Vienna 2, 07100 Sassari, Italy; rcappai1@uniss.it (R.C.)

Correspondence: maddalena.corsini@unisi.it (M.C.), nurchi@unica.it (V.M.N.)

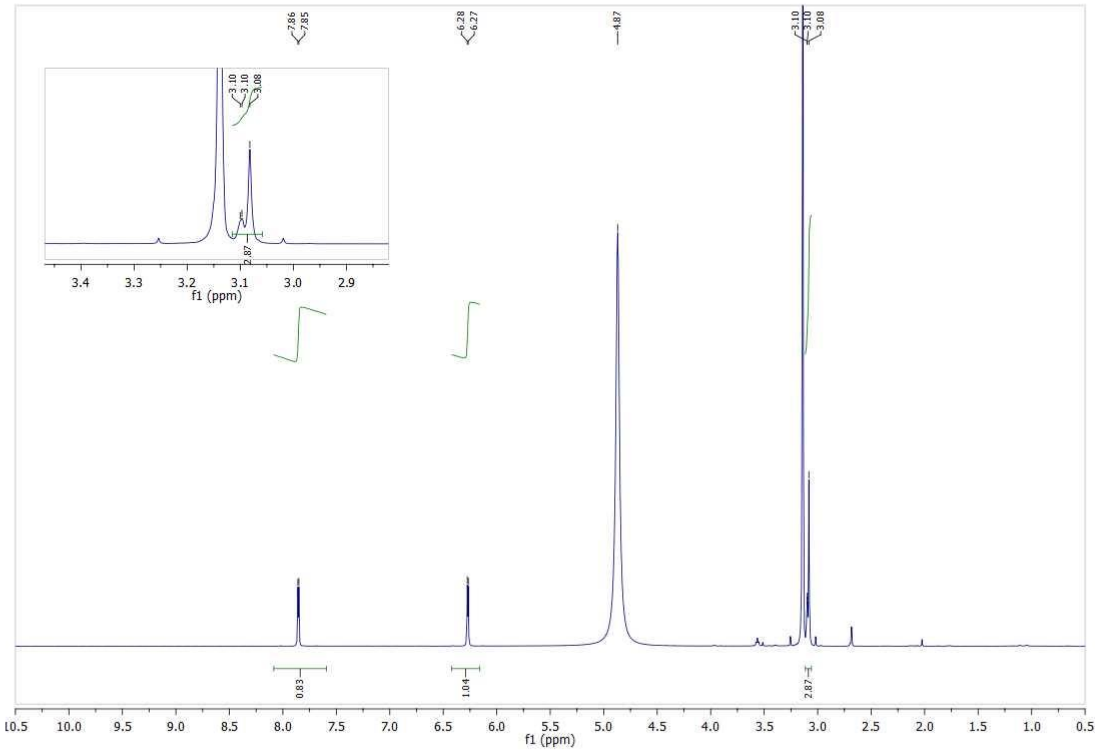

Figure S1. ^{1}H-NMR spectrum of 2-((3-hydroxy-4-oxo-4H-pyran-2-yl)methyl)malonic acid (**3**) in MeOD.

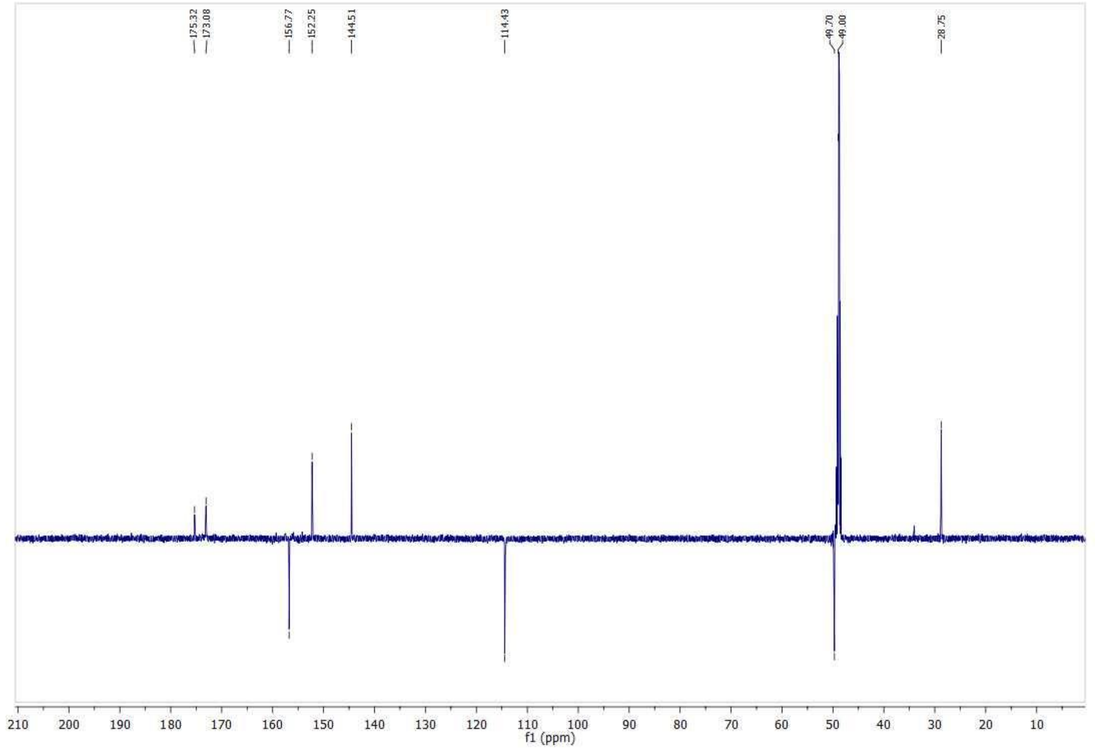

Figure S2. ^{13}C-NMR spectrum of 2-((3-hydroxy-4-oxo-4H-pyran-2-yl)methyl)malonic acid (**3**) in MeOD.

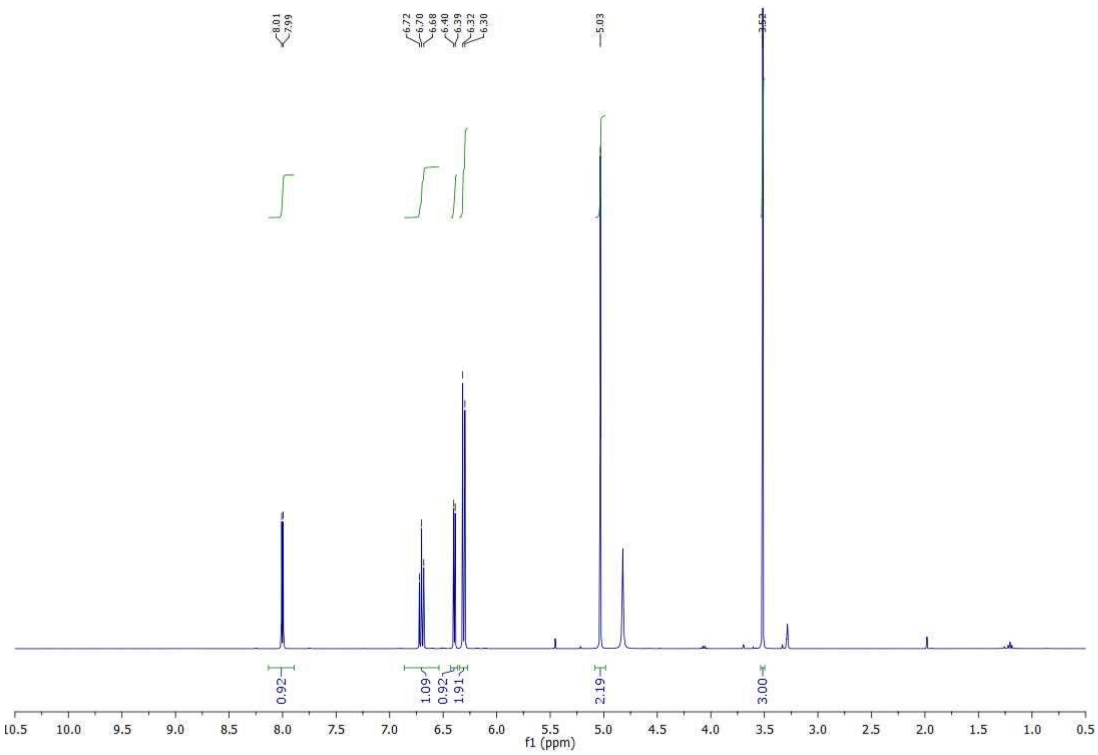

Figure S3. ^{1}H-NMR spectrum of 2-((2,6-dihydroxyphenoxy)methyl)-3-methoxy-4H-pyran-4-one (**4**) in MeOD.

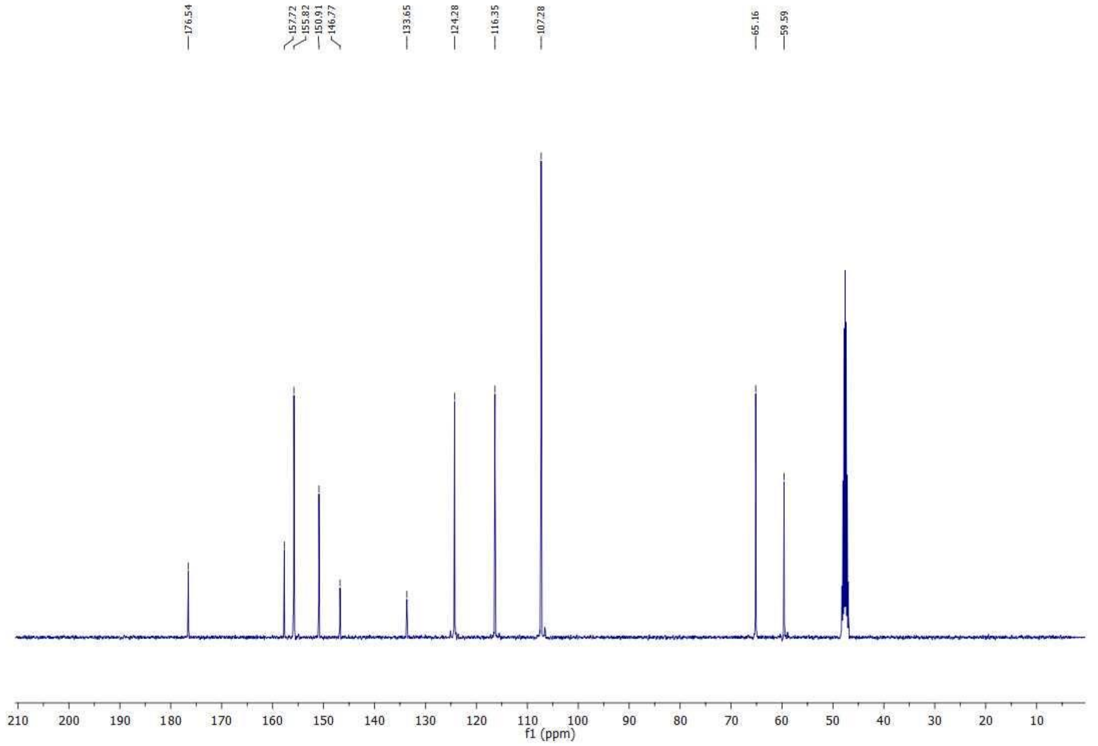

Figure S4. ^{13}C-NMR spectrum of 2-((2,6-dihydroxyphenoxy)methyl)-3-methoxy-4H-pyran-4-one (**4**) in MeOD.

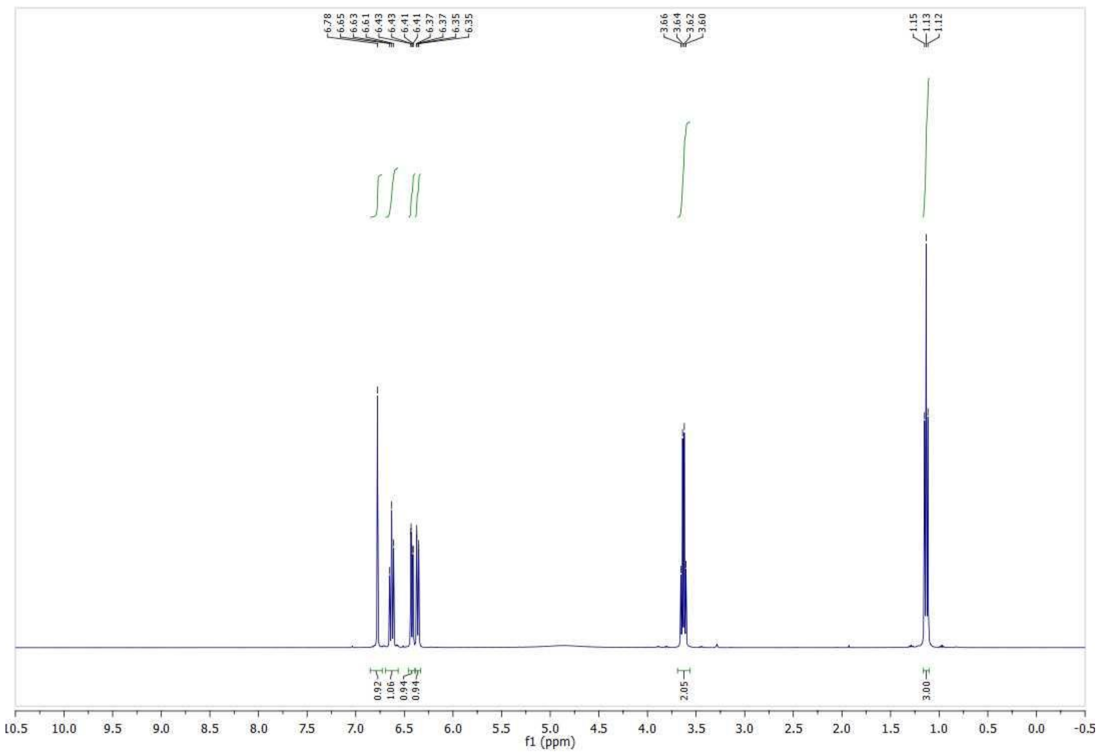

Figure S5. ^{1}H-NMR spectrum of 2-ethoxybenzo[d][1,3]dioxol-4-ol (**5**) in MeOD.

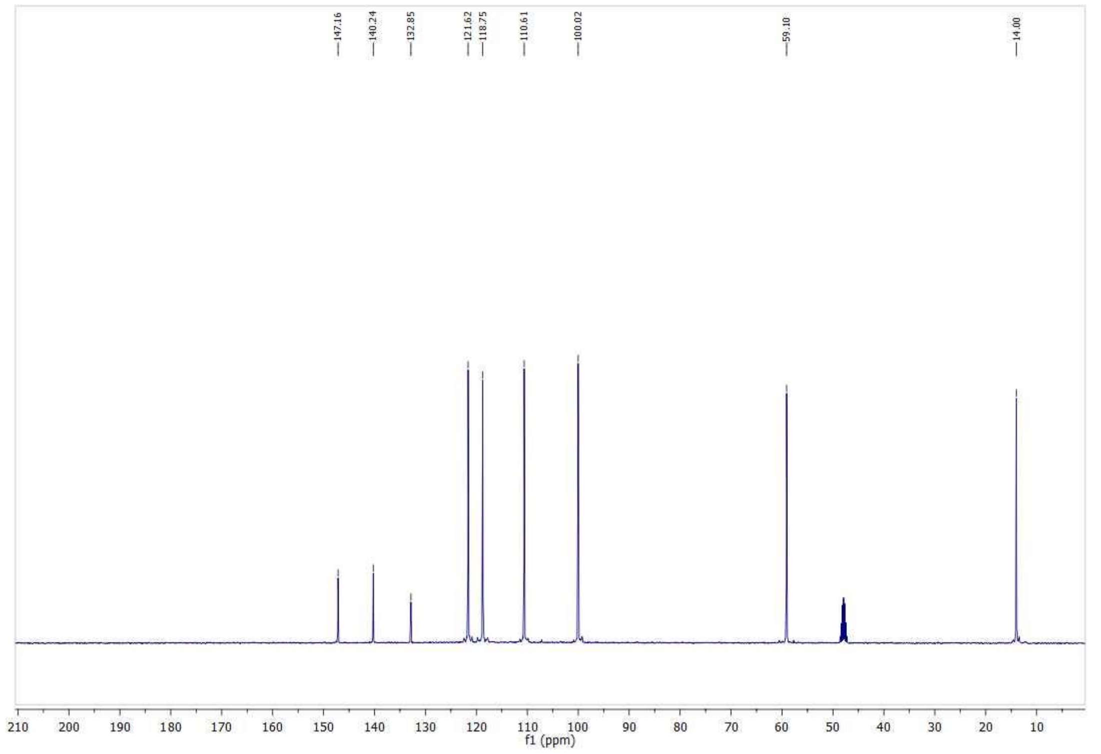

Figure S6. ^{13}C-NMR spectrum of 2-ethoxybenzo[d][1,3]dioxol-4-ol (**5**) in MeOD.

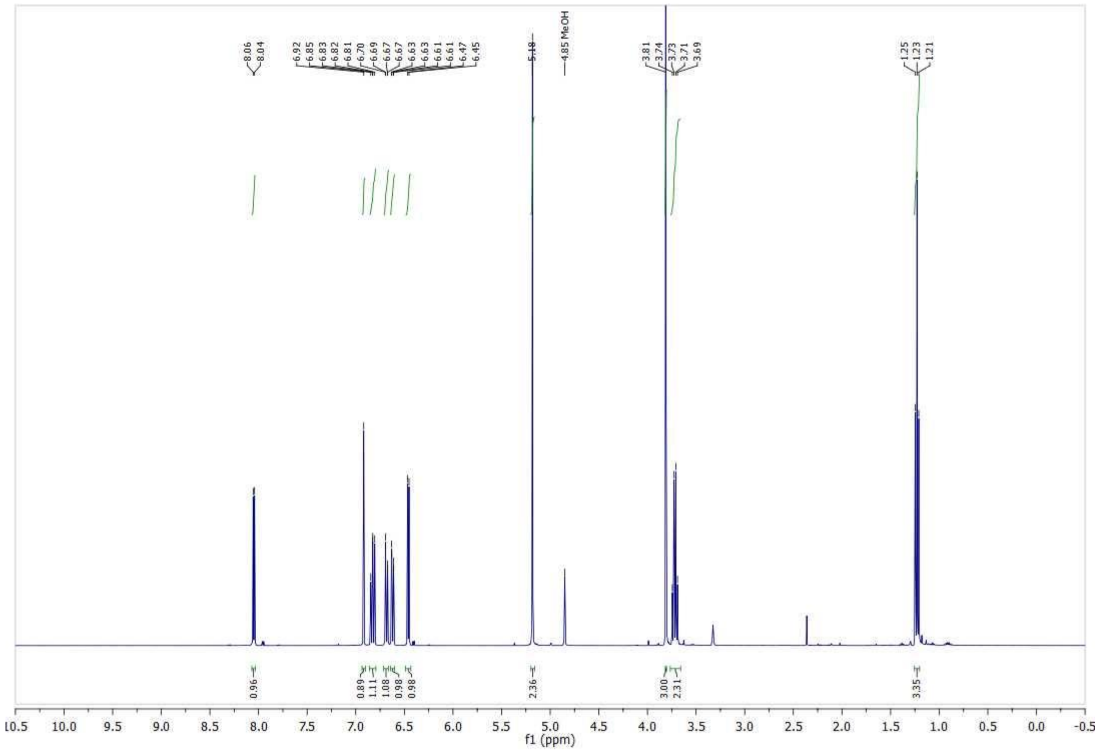

Figure S7. ^{1}H-NMR spectrum of 2-(((2-ethoxybenzo[d][1,3]dioxol-4-yl)oxy)methyl)-3-methoxy-4H-pyran-4-one (**6**) in MeOD.

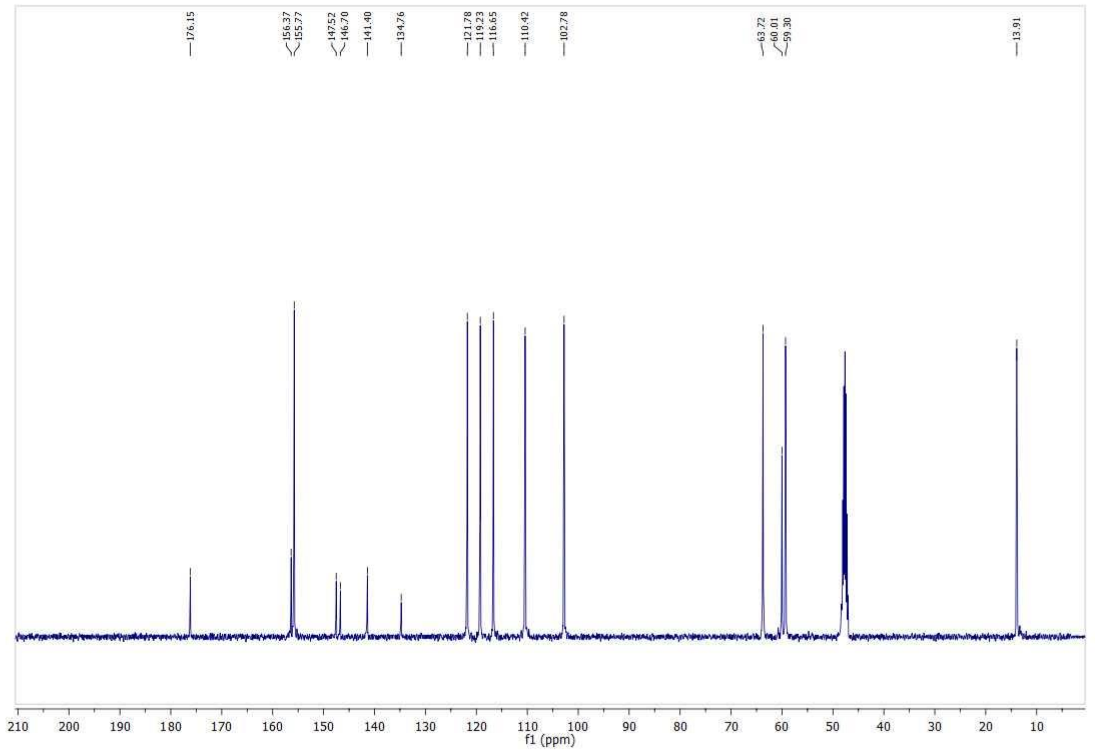

Figure S8. ^{13}C-NMR spectrum of 2-(((2-ethoxybenzo[d][1,3]dioxol-4-yl)oxy)methyl)-3-methoxy-4H-pyran-4-one (**6**) in MeOD.

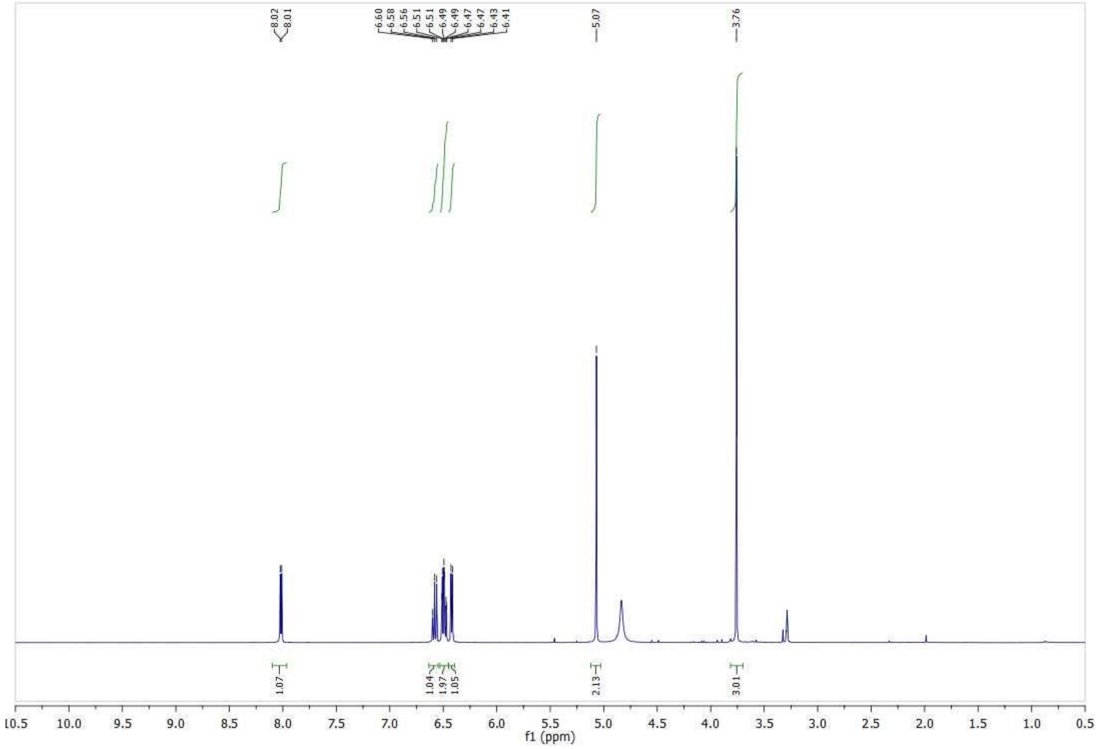

Figure S9. ^{1}H-NMR spectrum of 2-((2,3-dihydroxyphenoxy)methyl)-3-methoxy-4H-pyran-4-one (**7**) in MeOD.

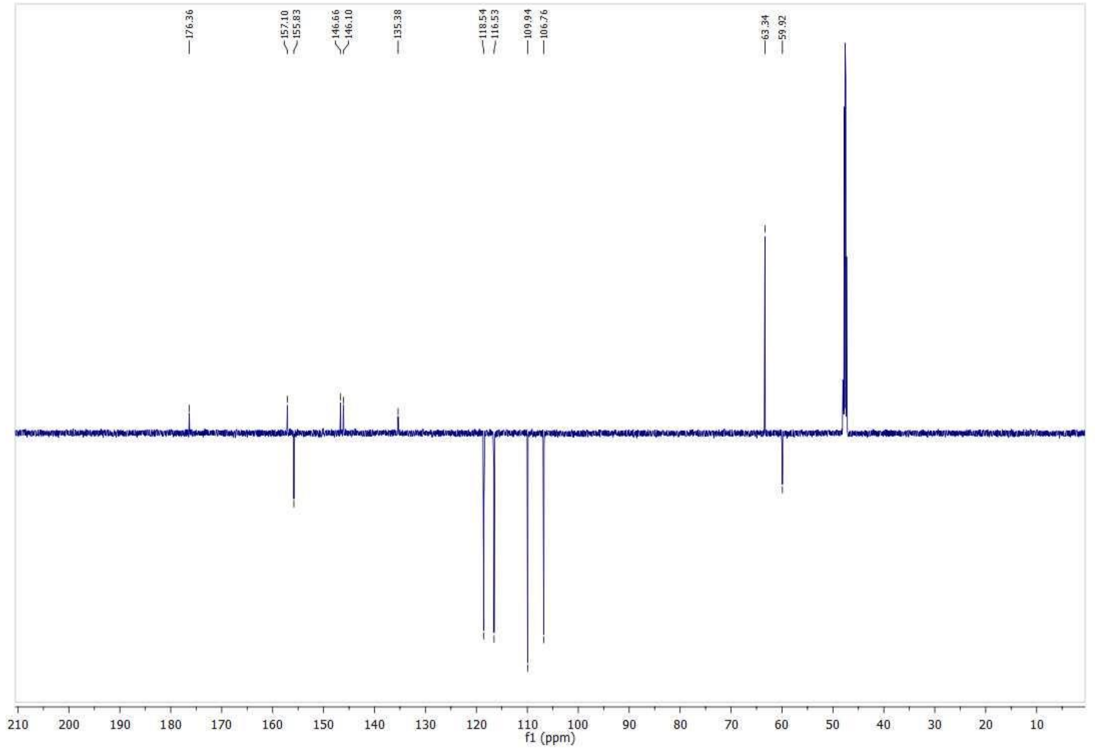

Figure S10. ^{13}C-NMR spectrum of 2-((2,3-dihydroxyphenoxy)methyl)-3-methoxy-4H-pyran-4-one (**7**) in MeOD.

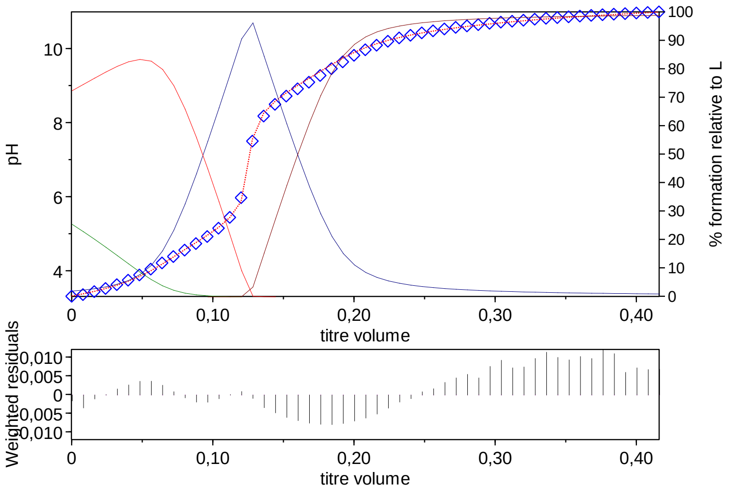

Figure S11. Potentiometric titration of **3** ligand at 25°C, 0.1 NaCl ionic strength, 6 × 10^{-4} M ligand concentration and 3.34-11.03 pH range. Hyperquad screenshot. . Experimental results are reported as blue points, calculated ones as dotted red line.

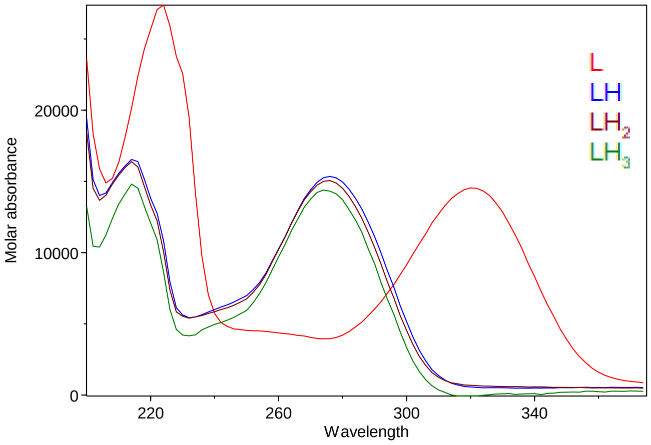

Figure S12. Molar absorptivity spectra of **3** ligand.

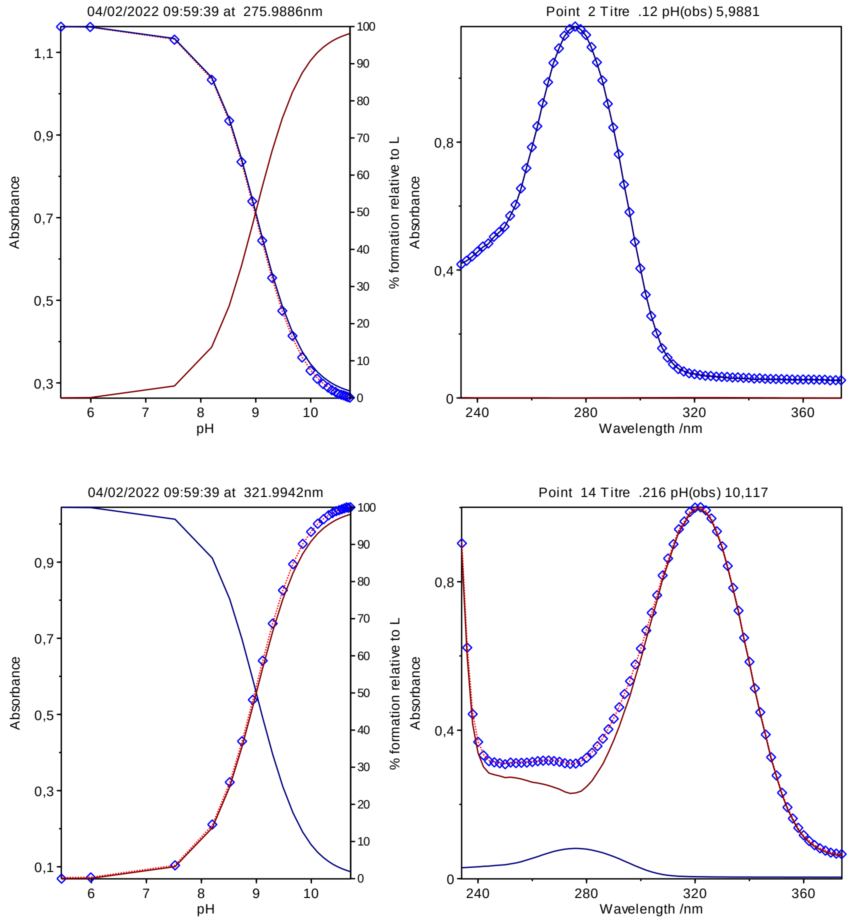

Figure S13. UV titration data of **3** ligand at 6.3 × 10^{-4} M ligand concentration: trend of absorbance vs pH on the maxima of the deprotonated form L^{3-} at 322 nm (bottom) and of the protonated form LH^{2-} at 276 nm (top), and spectra at pH 10.12 (bottom) and 5.99 (top). HypSpec screenshot. Experimental results are reported as blue points, calculated ones as dotted red lines.

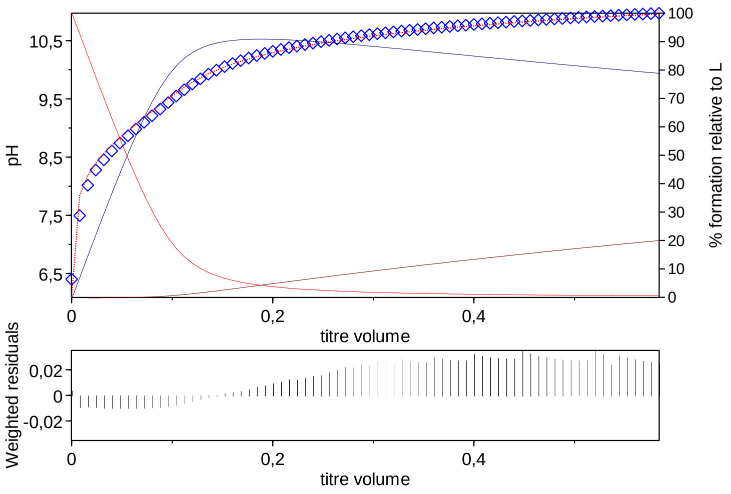

Figure S14. Potentiometric titration of **4** ligand at 25°C, 0.1 NaCl ionic strength, 5 × 10^{-4} M ligand concentration and 6.41-12.00 pH range. Hyperquad screenshot. . Experimental results are reported as blue points, calculated ones as dotted red lines.

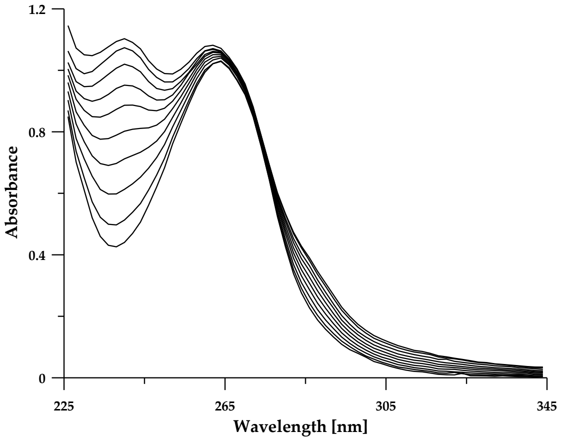

Figure S15. Representative spectra collected during UV titration of **4** ligand at 25°C, 0.1 NaCl ionic strength, 5 × 10^{-4} M ligand concentration, 225 – 345 wavelength range and 6.41 – 12.00 pH range.

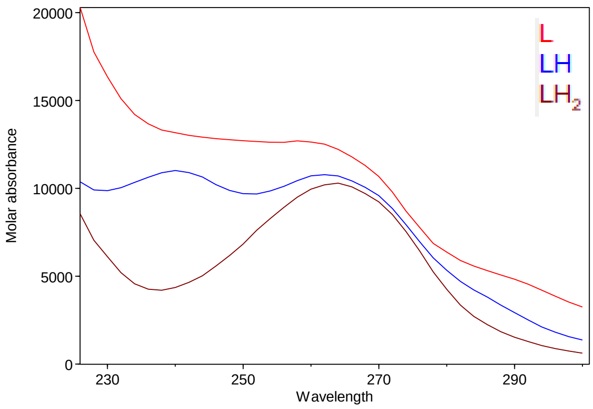

Figure S16. Molar absorptivity spectra of **4** ligand.

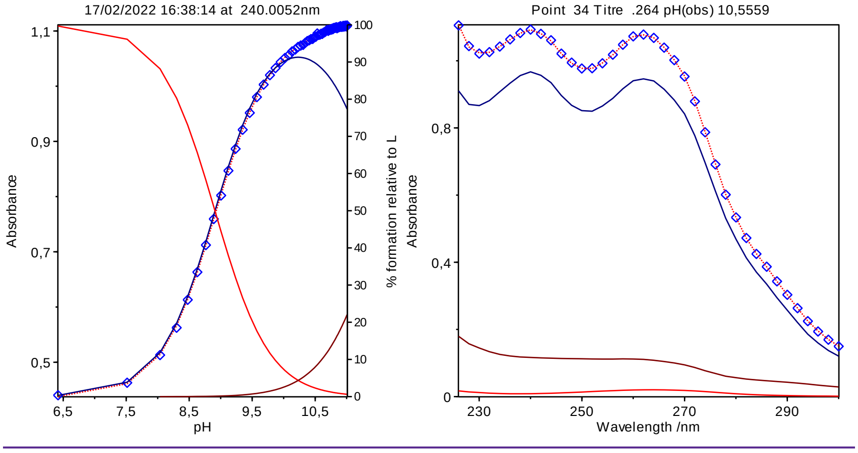

Figure S17. UV titration data of 4 ligand at 5×10^{-4} M ligand concentration: trend of absorbance vs pH at 240 nm where the major variation of absorbance is observed. HypSpec screenshot. Experimental results are reported as blue points, calculated ones as dotted red lines.

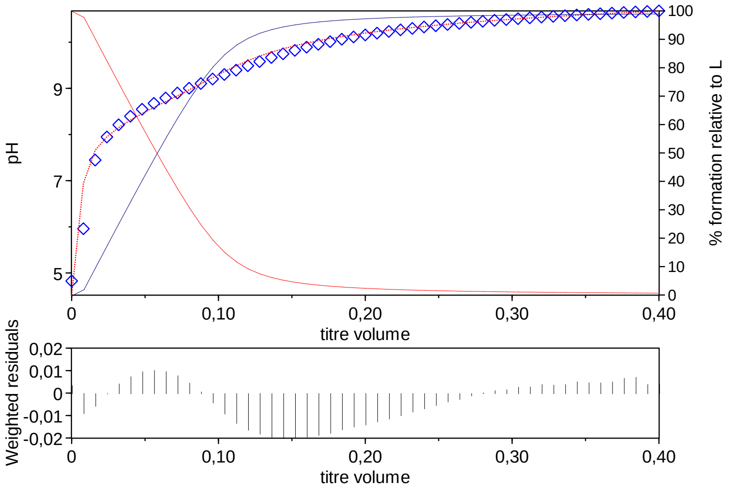

Figure S18. Potentiometric titration of **7** ligand at 25°C, 0.1 NaCl ionic strength, 5 × 10^{-4} M ligand concentration and 4.84-12.00 pH range. Hyperquad screenshot. . Experimental results are reported as blue points, calculated ones as dotted red lines.

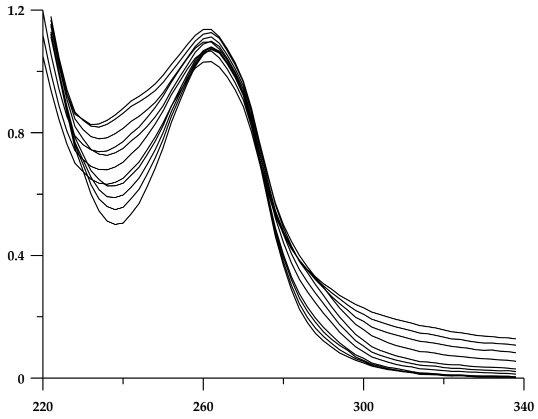

Figure S19. Representative spectra collected during UV titration of **7** ligand at 25°C, 0.1 NaCl ionic strength, 5 × 10^{-4} M ligand concentration, 220 – 340 nm wavelength range and 4.84 – 12.00 pH range.

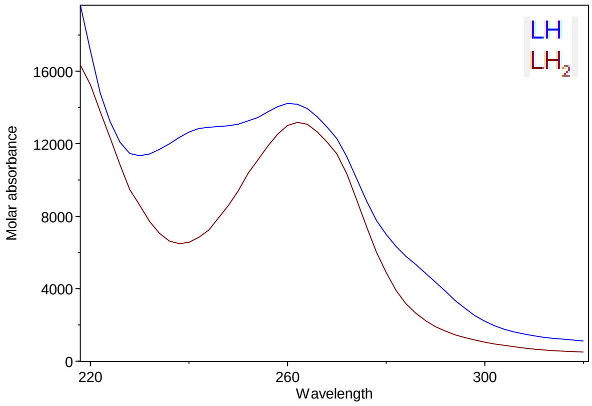

Figure S20. Molar absorptivity spectra of **7** ligand.

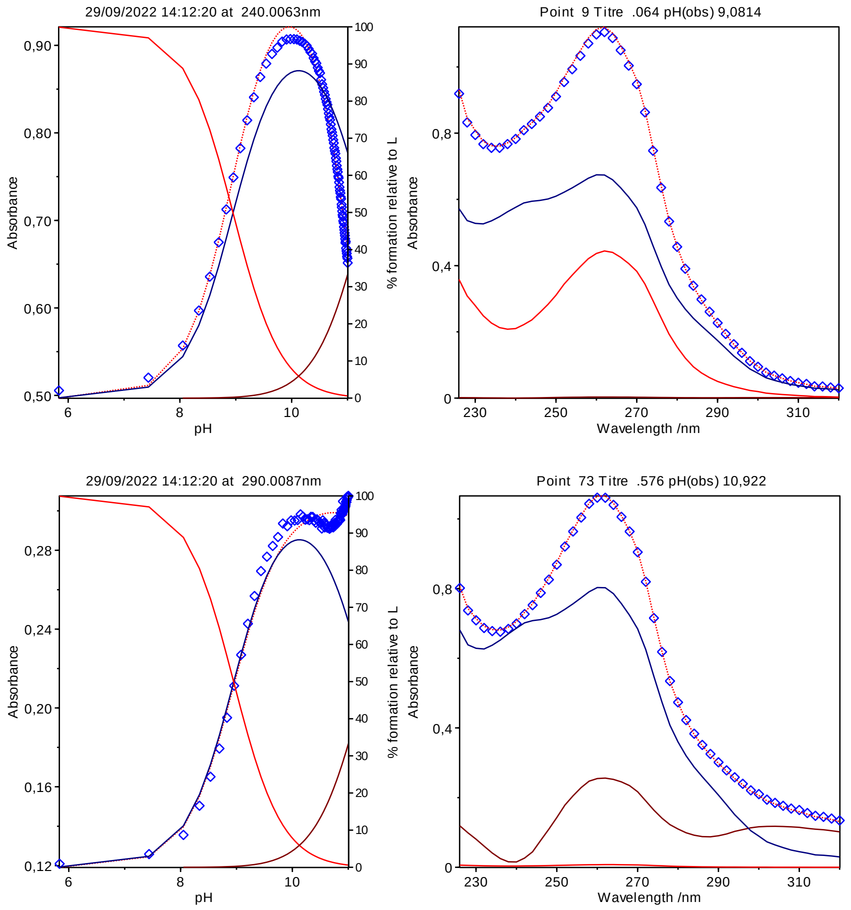

Figure S21 UV titration data of **7 l**igand at 5.0 × 10^{-4} M ligand concentration: trend of absorbance vs pH at the two representative wavelengths 240 nm (top) and 290 nm (bottom). Spectra at pH 9.08 (top) and 10.92 (bottom). HypSpec screenshots. Experimental results are reported as blue points, calculated ones as dotted red lines.

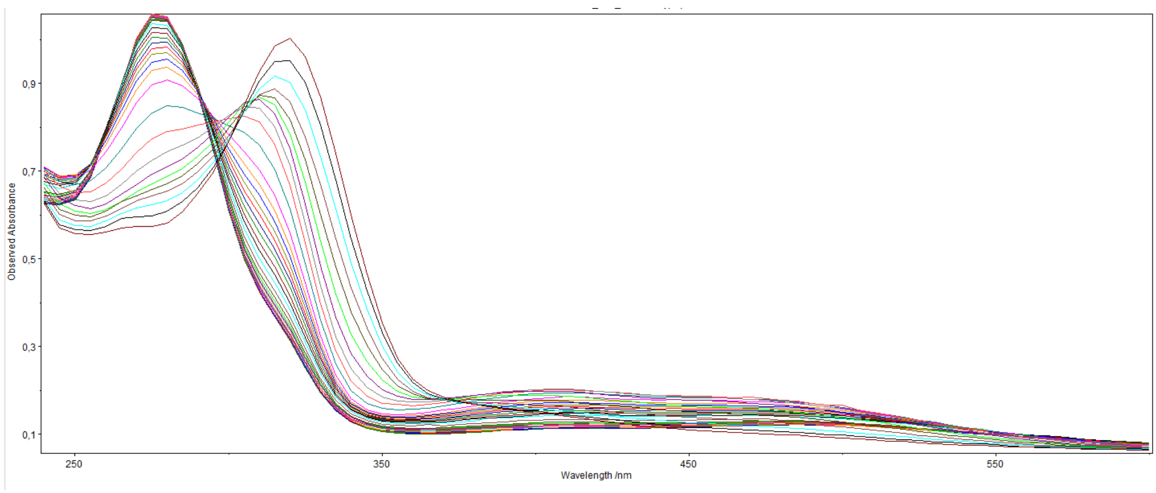

Figure S22. Spectra collected during UV-Vis titration of Fe^{3+}-**3** system at 25°C, 0.1 NaCl ionic strength, 1:3 metal:ligand molar ratio, 6.4 × 10^{-4} M ligand concentration, 240 – 600 wavelength range and 2.95 – 9.65 pH range.

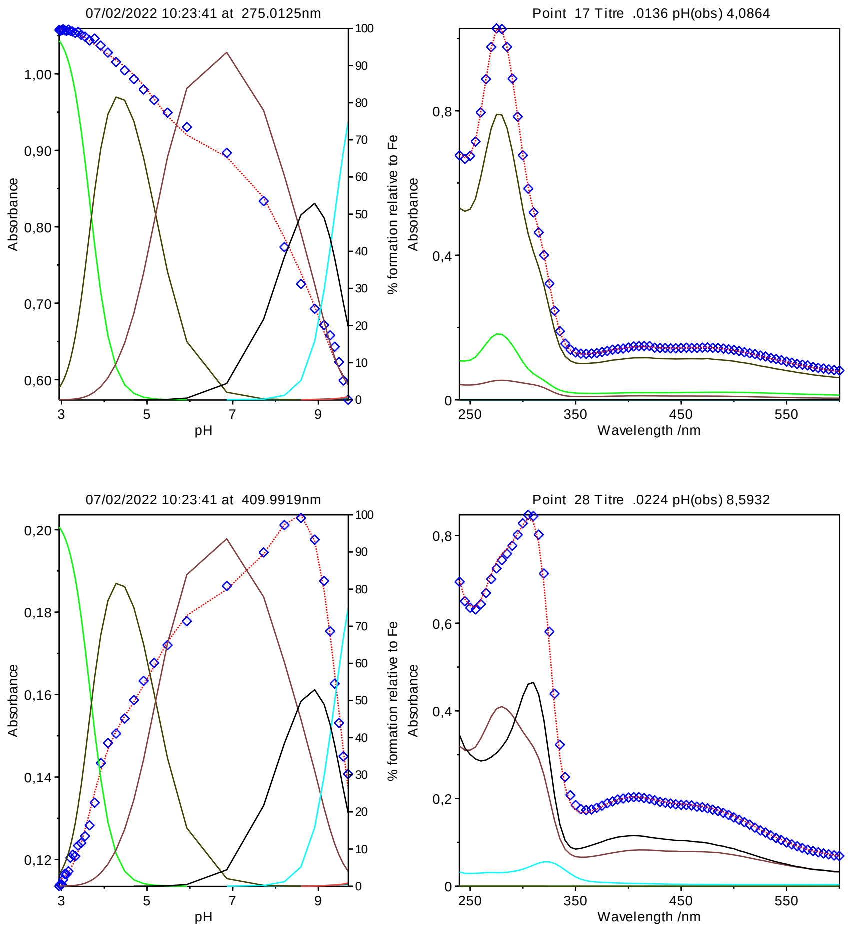

Figure S23. Spectra collected during UV-Vis titration of Fe^{3+}-**3** system at 25°C, 0.1 NaCl ionic strength: trend of absorbance vs pH at the two representative wavelengths 275 nm (top) and 410 nm (bottom). Spectra at pH 4.09 (top) and 8.59 (bottom). HypSpec screenshots. Experimental results are reported as blue points, calculated ones as dotted red lines.

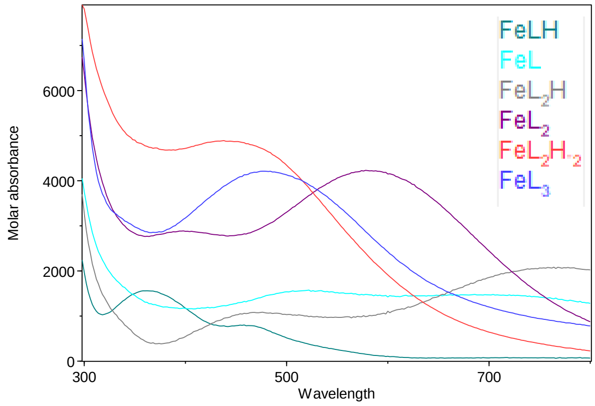

Figure S24. Molar absorptivity spectra of formed complexes in the Fe^{3+}-**7** system. HypSpec screen shot.

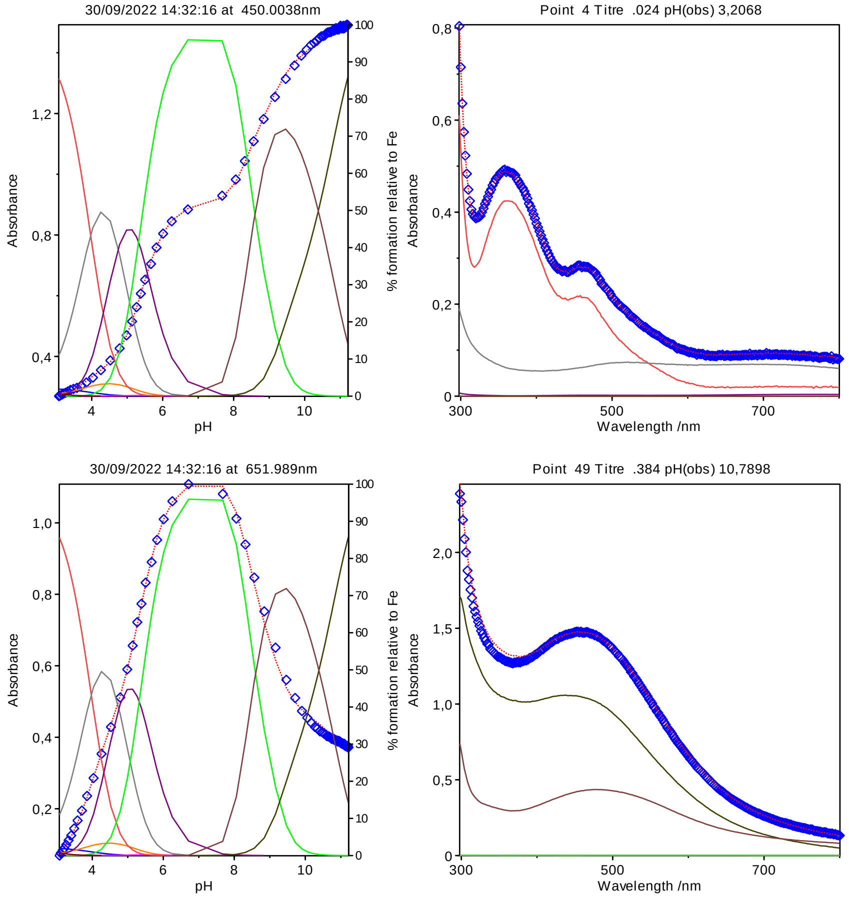

Figure S25. UV-Vis titration data of Fe^{3+}-**7** system at 1:3 metal:ligand molar ratio and 9.74 x 10^{-4} M ligand concentration: trend of absorbance vs pH at the two representative wavelengths 450 nm (top) and 652 nm (bottom), and two representative spectra at pH 3.20 (top) and 10.78 (bottom). HypSpec screenshots. Experimental results are reported as blue points, calculated ones as dotted red lines.

.

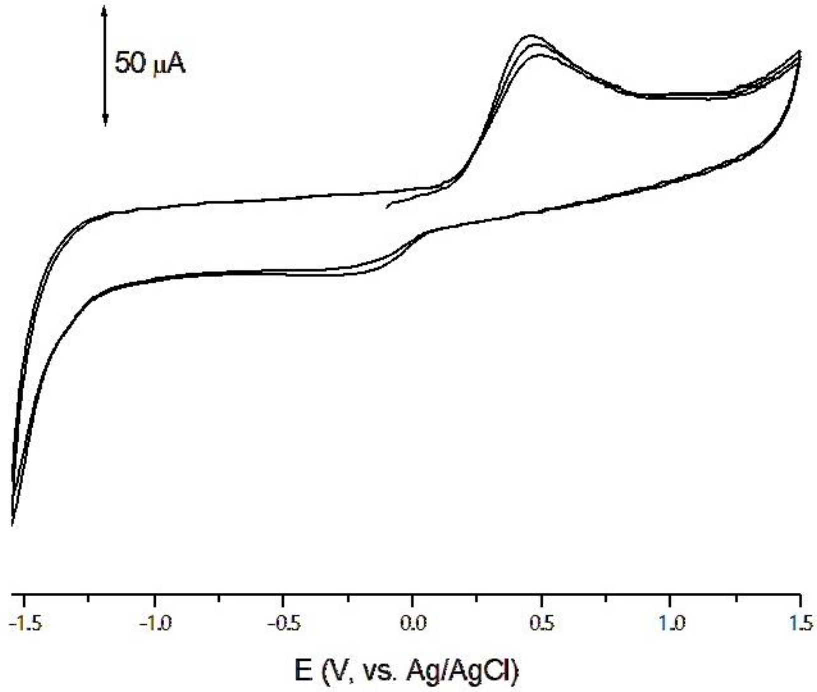

Figure S27. Cyclic voltammograms recorded on glassy carbon electrode on a solution 3 mM of **7** and $FeCl_3$ (3:1 molar) in buffer phosphate. Scan rate 200 mV/s

Table S1. Log K_1 and log K_2 of methyl malonic acid, malonic acid and maltol from literature reports.

**Methylmalonic acid**			**Malonic acid**			**Maltol**	
log K_1	log K_2		log K_1	log K_2		log K_1	
5.40	2.94	[31]	5.290	2.635	[33]	8.51	[34]
5.35	2.89	[32]				8.62	[35]

[31] Ostacoli, G.; Vanni, A.; Roletto, E. Complex formation between alkyl-substituted malonic acids and bivalent metal ions in aqueous solutions. *Ric. Sci.* **1968**, *38*, 318–321.
[32] Amico, P.; Bonomo, R.P.; Cali, R.; Cucinotta, V.; Daniele, P.G.; Ostacoli, G.; Rizzarelli, E. Ligand-ligand Interactions in Metal Complexes: Thermodynamic and Spectroscopic Investigation of Simple and Mixed Copper(II) and Zinc(II) Substituted-malonate Complexes with 2,2'-bipyridyl in Aqueous Solution. *Inorg. Chem.* **1989**, *28*, 3555–3561, doi:10.1021/ic00317a032.
[33] DeRobertis, A.; DeStefano, C.; Foti, C. Medium effects on the protonation of carboxylic acids at different temperatures. *J. Chem. Eng. Data* **1999**, *44*, 262–270, doi:10.1021/je980239j.
[34] Petrola, R. Spectrophotometric study on the equilibria of pyromeconic acid derivatives with proton in aqueous solution. *Finnish Chem. Lett.* **1985**, *12.5*, 207
[35] Chiaccherini, E.; Bartušek, M. Complexes of maltol with uranyl and aluminium. *Collec. Czech. Chem. Commun.* **1969**, *34*, 530-536, doi:10.1135/cccc19690530.
